# Energetics of carboxylate-metal bonds in polymetallic rings†

**DOI:** 10.1039/d5cc01911g

**Published:** 2025-06-11

**Authors:** Niklas Geue, Tim Renningholtz, George F. S. Whitehead, Grigore A. Timco, Cristina Trujillo, Perdita E. Barran, Richard E. P. Winpenny

**Affiliations:** a Michael Barber Centre for Collaborative Mass Spectrometry, Manchester Institute of Biotechnology, Department of Chemistry, The University of Manchester 131 Princess Street Manchester M1 7DN UK niklas.geue@manchester.ac.uk perdita.barran@manchester.ac.uk; b Department of Chemistry, The University of Manchester Oxford Road Manchester M13 9PL UK richard.winpenny@manchester.ac.uk

## Abstract

Using collision-induced dissociation mass spectrometry and density functional theory, we assess the energetics of polymetallic [Cr_7_NiF_8_(O_2_CR)_16_]^−^ anions *in vacuo*, showing how the carboxylate influences the ring stability. We find the best correlation with the enthalpic contribution to the computed proton affinity of the isolated carboxylates, demonstrating how the stability of polymetallic rings can be predicted *in silico*.

## Abstract 2

Using collision-induced dissociation mass spectrometry and density functional theory, we assess the energetics of polymetallic [Cr_7_NiF_8_(O_2_CR)_16_]^−^ anions *in vacuo*, showing how the carboxylate influences the ring stability.

## Graphical abstract

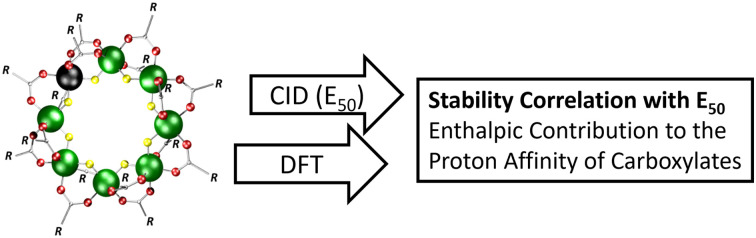

The design of polymetallic complexes has attracted considerable attention over the last decades, with such compounds studied for potential applications in catalysis, extraction, as well as quantum materials.^1–4^ Their synthesis often relies on self-assembly reactions, leading to the formation of energetically stable 2D and 3D structures with fascinating properties.^2,3^ Due to the structural complexity of the complexes and their building blocks, factors governing their stability have rarely been studied which limits a systematic exploitation of these metallosupramolecules.

Mass spectrometry (MS) methods have emerged as powerful tools to understand the structure and properties of large synthetic assemblies, and particularly collision-induced dissociation mass spectrometry (CID-MS) played a major role in this advancement.^5–7^ In CID-MS, ions of interest are isolated through their mass-to-charge (*m*/*z*) ratio in a quadrupole mass filter, and subsequently subjected to collisions with an inert gas at user-defined energies.^8^ These collisions commonly induce structural changes and/or fragmentation to smaller ions and neutrals, and the stoichiometry of the fragments, as measured by their mass, can inform on the connectivity and structure of the precursor ion.^6^ CID-MS is also a powerful tool to measure ions’ energetics, which can be quantified through a collision energy ramp. Monitoring the fragment ion intensities at different collision energies can give rise to the median collision energy that is required for fragmentation (*E*_50_ value, see below), which is comparable across similar ions.^9,10^

We have been interested in the design of polymetallic chromium rings for over two decades.^1^ These molecules have potential applications in quantum computing,^11^ or in host–guest chemistry^12^ and show promise as lithographic resists.^13^ Particularly for the latter, where rapid decomposition of the metal–organic compound is key to high sensitivity of the resist, an understanding of complex stability is important.

From our family of polymetallic rings, the most fundamental structure is [Cr_7_M^II^F_8_(O_2_CR)_16_]^−^, in which seven Cr^III^ and one M^II^ centre are arranged in an octagon, and bridged *via* two carboxylate ligands and one fluoride on each edge (Fig. 1 for M^II^ = Ni^II^).^1^ Previously, we have shown that the stability of these {Cr_7_M^II^}^−^ rings strongly depend on the divalent metal M^II^, and the trends followed arguments from crystal field theory (for 2^−^ and isostructural {Cr_7_M^II^}^−^ rings with other divalent metals M^II^).^14^ The {Cr_7_M^II^}^−^ anions can also encapsulate ammonium cations [NH_2_XX′]^+^, and the disassembly of these (pseudo)rotaxanes was shown to depend on steric and electronic effects of the stopper groups X and X′,^14,15^ as well as the charge carrier ions used for ionization.^16^ For related complexes of the same family, we also investigated the impact of the complex topology.^17^

**Fig. 1:**
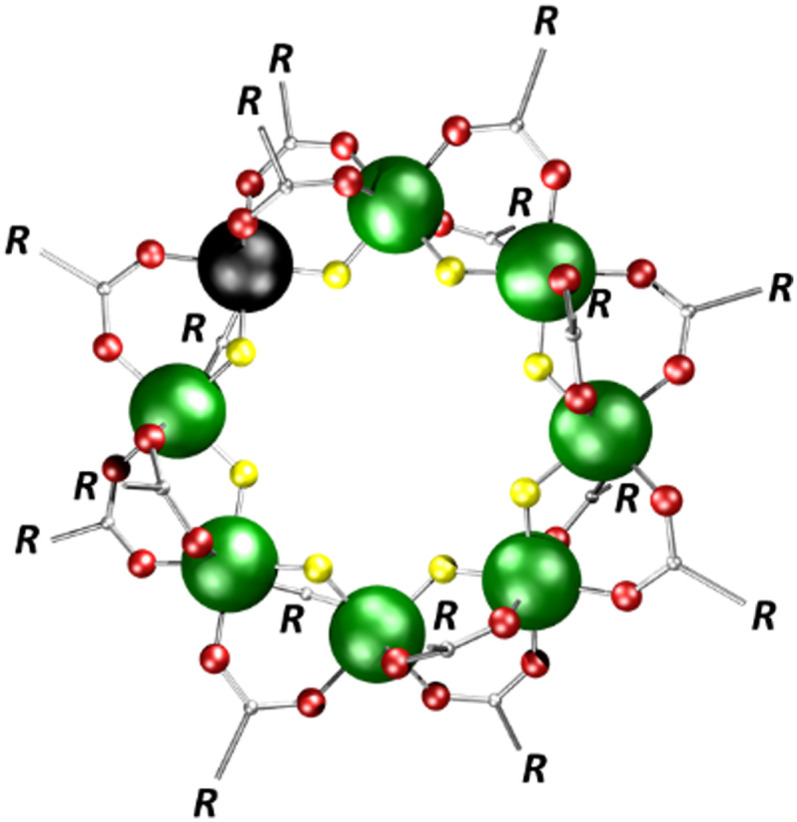
Structure of the polymetallic ring [Cr_7_Ni^II^F_8_(O_2_CR)_16_]^−^. (Cr: green, Ni: black, F: yellow, O: red, C: grey).

Here, we quantify the stability of thirteen different [Cr_7_M^II^F_8_(O_2_CR)_16_]^−^ ions using CID-MS, showing that R has a strong control over the stability of polymetallic complexes. The correlation of their *E*_50_ values with various computed molecular and atomistic descriptors for the carboxylates/carboxylic acids yielded the best agreement with the enthalpic contribution to the simulated proton affinity of the carboxylic acids. This is a step towards being able to predict the stability of polymetallic complexes with confidence. Experimental details can be found in the ESI.†

The gaseous [Cr_7_M^II^F_8_(O_2_CR)_16_]^−^ rings (1^−^–13^−^, with different carboxylic acids as reagents, shown in Fig. 2) were obtained from solutions of [Cat][Cr_7_M^II^F_8_(O_2_CR)_16_] (where Cat^+^ = NH_2_^*n*^Pr_2_^+^ for 1^−^, 2^−^, 4^−^, 7^−^–12^−^; NH_3_^*n*^Pr^+^ for 3^−^; and NH_2_(CH_2_=CH–CH)_2_^+^ for 5^−^ and 6^−^). Fig. S1 (ESI†) illustrates the mass spectrum of [NH_2_^*n*^Pr_2_][2] in negative mode including the isotopic distribution for 2^−^. 1^−^–13^−^ were isolated in a quadrupole according to their *m*/*z*-ratio and subjected to collisional activation at different energies. All fragment in a similar manner and show the loss of (i) [Ni(O_2_CR)_2_], (ii) [Cr(O_2_CR)_3_] and (iii) [Cr(O_2_CR)_2_F] leaving groups in the first step (Fig. 3a for fragmentation of 2^−^). The intensities of these fragments vary slightly, however the loss of [Ni(O_2_CR)_2_] is in all cases but one the dominant disassembly channel. The exception is 7^−^, where the loss of [Cr(O_2_CR)_3_] is the dominant fragmentation channel (Fig. S2, ESI†). The fragmentation behaviour of 13^−^ is unusual in a different way, with additional, minor loss of CO_2_ in different dissociation steps (Fig. S3, ESI† including the isotopic pattern for [13^−^–CO_2_]^−^).

**Fig. 2:**
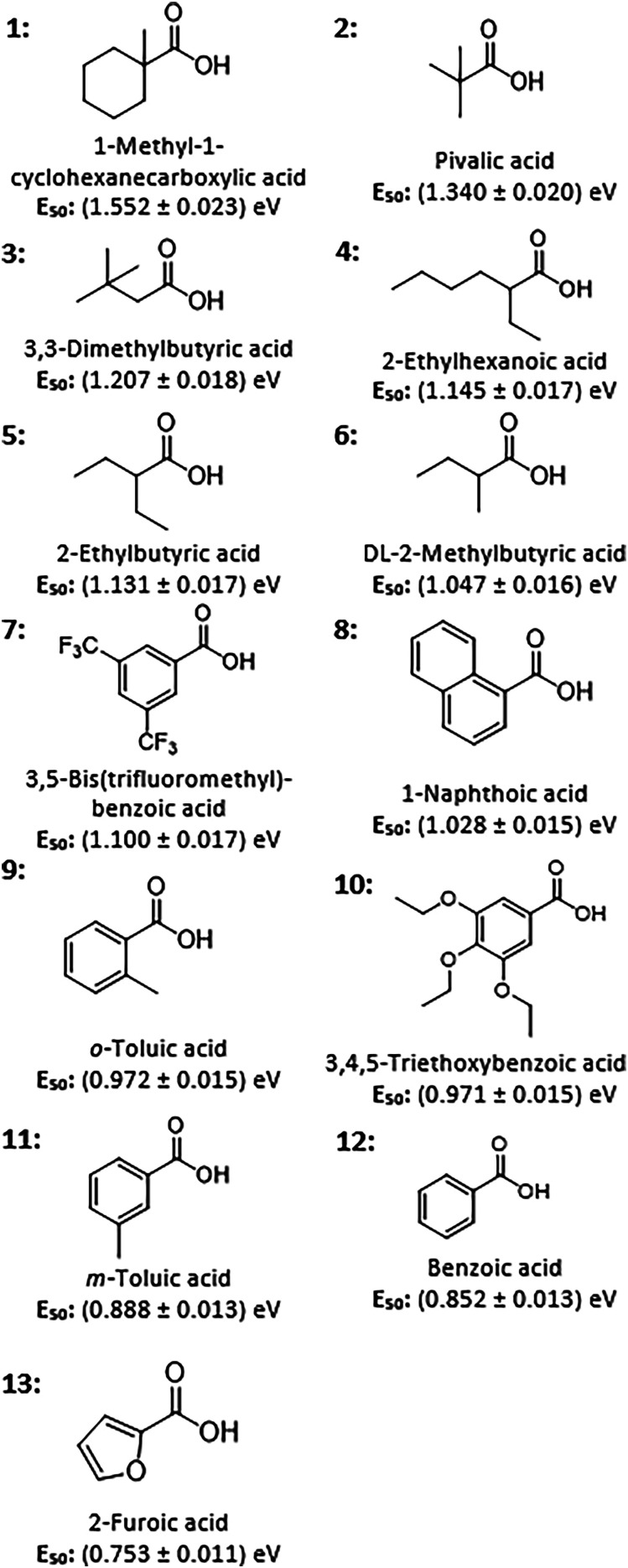
Carboxylic acids used in their deprotonated form for the studied polymetallic ring anions 1^−^–13^−^ and the rings’ *E*_50_ values. ^*a*^ This value varies slightly from the previously published *E*_50_ value, however the difference is within 1.5%, agreeing with the error given.^14^

**Fig. 3:**
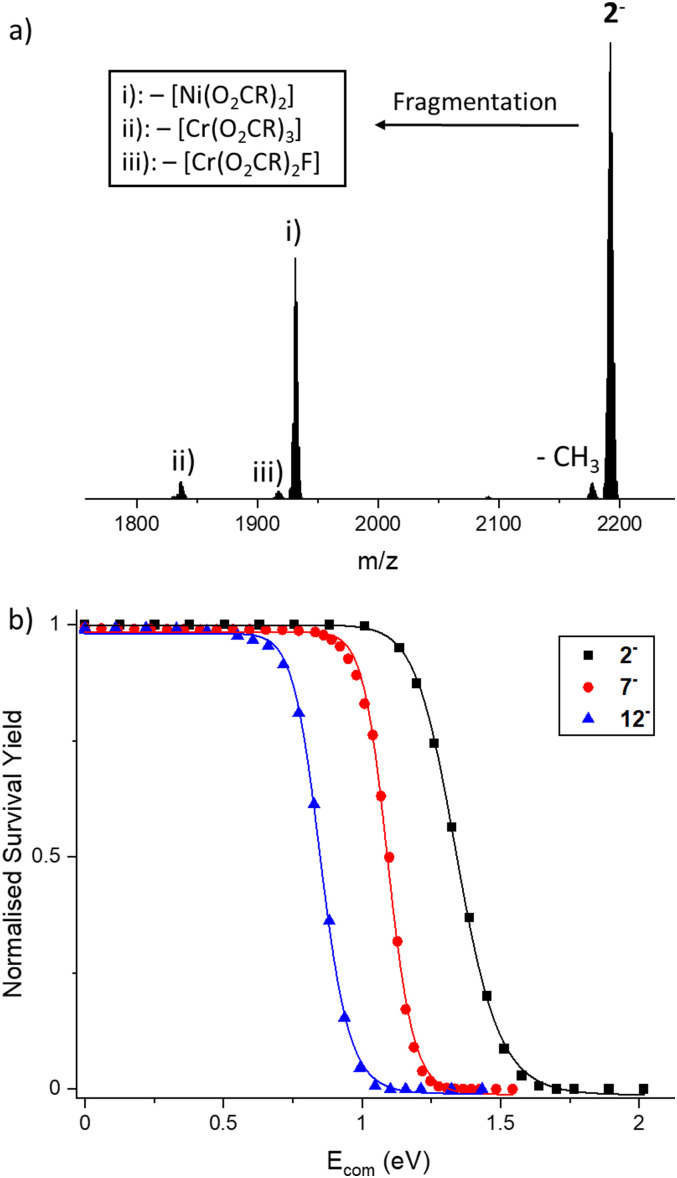
(a) CID-MS spectrum of 2^−^ (*m/z* = 2192) at *E*_lab_ = 105 eV. Besides the metastable loss of a methyl group from the carboxylate ligands, the three fragments (i), (ii) and (iii) were found that correspond to the losses of [Ni(O_2_CR)_2_], [Cr(O_2_CR)_3_] and [Cr(O_2_CR)_2_F], respectively. (b) Normalized survival yield *vs. E*_com_ fitted to a sigmoidal Hill function, exemplary for 2^−^, 7^−^ and 12^−^. The three ions follow the stability (and *E*_50_ value) order of 2^−^ > 7^−^ > 12^−^ (Fig. 2).

For each ring, the signal intensity of the parent ion was tracked across the collision energy ramp, relative to the summed intensities of all fragment ions and the parent ion. This leads to the so-called “survival yield” (SY), and plotting SY as a function of collision energy in the centre-of-mass frame (*E*_com_) results in sigmoidal curves (Fig. 3b and Fig. S4–S13, ESI†). The transition point, at which 50% of the parent ion fragments, was quantified through a fitting function. This *E*_50_ value is regarded as a relative measure of ion stability.^9,10^*E*_50_ values were recorded for all ring anions (Fig. 2). In general, they depend on pressure and voltages in the collision cell, as well as the inert gas (here: N_2_). Other factors, such as capillary voltage, source temperature, solution conditions, in-source trapping voltages were hypothesised to have no effect. For 2^−^ several controls were performed that all yielded highly reproducible values, with all of them within 1.5% (Supplementary Dataset, ESI†). Hence, an error of 1.5% was defined for all *E*_50_ values (Fig. 2).

The *E*_50_ values show differences of *ca.* 106% between the most stable anion 1^−^ (*E*_50_ = 1.552 eV) and the least stable 13^−^ (*E*_50_ = 0.753 eV), suggesting that R significantly influences the stability of the ring anion. This variation is considerably higher than those due to changes in metal composition,^14^ adduct ions,^16^ amine cations^14,15^ and ring topologies.^17^

Overall, aliphatic carboxylates are more stable than aromatic ligands, however large differences are observed within each group (Fig. 2). We hypothesised that the properties of the free carboxylic acids/carboxylates might determine the relative stability differences between the ring anions. This would enable a computationally inexpensive prediction of polymetallic ring stability *in silico*, facilitating the design of such complexes. Density functional theory (DFT) was used to model several properties, and correlations with the *E*_50_ value were found for the proton affinity (PA), p*K*_A_ value (in water), the Weizsäcker kinetic energy of the COO^−^ group in each carboxylate, and the wavenumber of the O–C–O asymmetric stretching vibration (Table S1, ESI†).

The relationship of *E*_50_ values with PA and p*K*_A_ values follow the argument that more acidic carboxylic acids (lower PA, lower p*K*_A_) result in less basic carboxylates and hence weaker binding to electrophiles such as H^+^, or in the case of the polymetallic rings, Ni^II^ and Cr^III^. This trend was indeed found for the PA of 1^−^–13^−^, however only with *R*^2^ = 0.33 after excluding the outlier 7^−^ (Fig. S14, ESI†). The correlation with the calculated pK_A_ is slightly stronger than the one with PA (*R*^2^ = 0.59 with 7^−^ as outlier, Fig. S15, ESI†). The best correlation is found when only considering the thermal and zero-point energy contributions, Δ*E*_cor_, to the PA and neglecting the electronic energy (Fig. 4, see ESI† for definition of Δ*E*_cor_). The dominant term in Δ*E*_cor_ is the change in zero-point energy upon protonation of the carboxylate. It can thus be understood as a measure for the O–H bond strength, where a high Δ*E*_cor_ indicates a strong O–H bond. This correlates with the stability of the Ni–O and Cr–O bonds (Fig. 4).

**Fig. 4:**
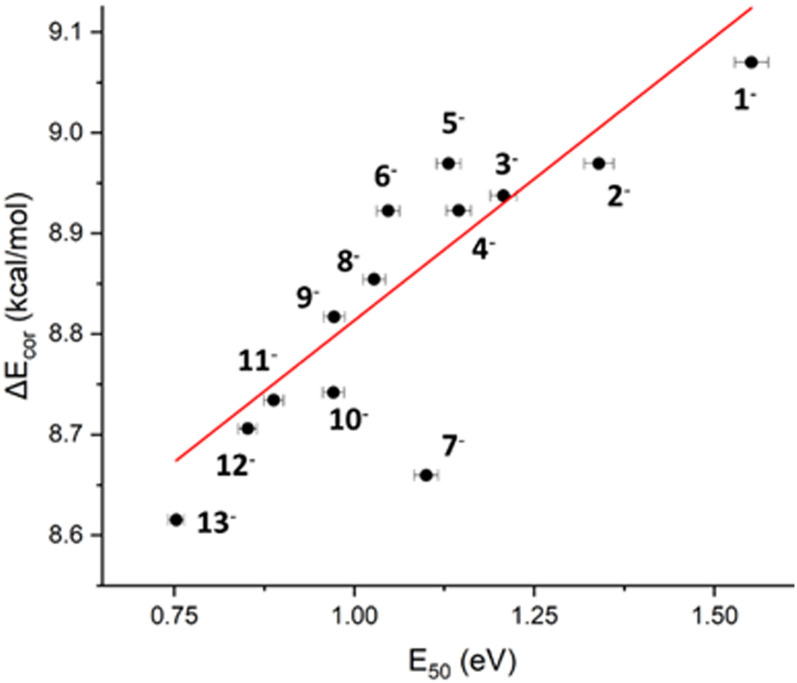
Plot of the enthalpic contributions, Δ*E*_cor_, to the proton affinity computed for RCO_2_^−^/RCO_2_H *vs. E*_50_ values. *R*^2^ = 0.86 when excluding the outlier 7^−^.

In addition to this thermodynamic term, we found a trend between an electron density proxy measure (Weizsäcker kinetic energy) at the carboxylate group and the *E*_50_ values, with a higher electron density correlated with more stable metal complex (Fig. S16, *R*^2^ = 0.71, ESI†). Lower electron density in the carboxylate region will result in weaker bonds to electrophiles such as Ni and Cr, hence agreeing with the *E*_50_ trend.

This led us to the conclusion that the properties of the isolated carboxylate/carboxylic acid, *i.e.* proton affinity, electron density, vibrational frequencies (Fig. S17, ESI†), are the main factors determining the stability of the entire metal complex. As many factors influence the *E*_50_ value of an ion, namely the stability of precursor ion, fragment ions and neutral leaving group, as well as number of collisions, site of collision, considerably stronger correlations cannot be expected.

Notably, the ring 7^−^ is an outlier in most correlation plots (Fig. 4), appearing considerably more stable than predicted by DFT. When modelling the full polymetallic complex 7^−^, intramolecular F–π interactions were found between the axial as well as axial and equatorial ligands (Fig. S18, ESI†). These stabilising interactions, which can only occur in 7^−^ due to the presence of fluorides in the ligands, are likely the source for the increased stability of the complex. Thus, although in most cases the properties of the carboxylate determine the stability of the complex (relative to other carboxylates), exceptions exist for cases where the ligands (de-)stabilise intramolecular interactions as in the case of 7^−^. This demonstrates that CID-MS is a suitable tool for experimentally verifying the DFT stability predictions of such polymetallic rings, and we believe that the methodology presented here can be applied to understand metal–organic binding in metallosupramolecular complexes in general.

We thank Dr Selena Lockyer for assistance with figures as well as Dr Neil Burton and Prof. David Collison (all The University of Manchester), for helpful discussions. The authors acknowledge the European Research Council for funding the MS SPIDOC H2020-FETOPEN-1-2016-2017-801406, BBSRC for the research grant BB/X002403/1, Waters Corporation for their continued support of mass spectrometry research within the Michael Barber Centre for Collaborative Mass Spectrometry and the assistance provided by Research IT and the use of the Computational Shared Facility at The University of Manchester. T. R. thanks The University of Manchester for a PhD studentship. R. E. P. W. thanks the European Research Council for an Advanced Grant (ERC-2017-ADG-786734). The authors also thank the staff in the MS and Separation Science Facility in the Faculty of Science and Engineering, The University of Manchester, for their assistance.

## Conflicts of interest

There are no conflicts to declare.

## Supplementary Material

CC-061-D5CC01911G-s001

CC-061-D5CC01911G-s002

## Data Availability

Supporting data, containing the raw data of mass spectrometry measurements, were deposited on Figshare (DOI: https://figshare.com/articles/dataset/Supplementary_Dataset_for_the_Energetics_of_Carboxylate-Metal_Bonds_in_Polymetallic_Rings_/28704452?file=54619652). DFT data are available at ioChem-BD (DOI: https://doi.org/10.19061/iochem-bd-6-515). CCDC 2431792–2431798 contains the supplementary crystallographic data for the compounds reported in this paper (Table S2 and Fig. S19–S25, ESI†).
