# Supplementary material for: Energetics of carboxylate-metal bonds in polymetallic rings
Source: Chem Commun (Camb). 2025 Jun 11;61(59):11049–52. doi: 10.1039/d5cc01911g (PMC12184960; doi:10.1039/d5cc01911g)
Supplement: CC-061-D5CC01911G-s001 [file CC-061-D5CC01911G-s001.pdf]

Supplementary Information (SI) for Chemical Communications.
This journal is © The Royal Society of Chemistry 2025

Energetics of Carboxylate-Metal Binding in Polymetallic Rings

Niklas Geue[1,*], Tim Renningholtz,[2] George F. S. Whitehead,[2] Grigore A. Timco,[2] Cristina Trujillo,[2] Perdita E. Barran[1,*] and Richard E. P. Winpenny,[2,*]

[1]Michael Barber Centre for Collaborative Mass Spectrometry, Manchester Institute of Biotechnology, Department of Chemistry, The University of Manchester, 131 Princess Street, Manchester, M1 7DN, UK. [2]Department of Chemistry, The University of Manchester, Oxford Road, Manchester, M13 9PL, UK.

[*]Corresponding Author: niklas.geue@manchester.ac.uk, perdita.barran@manchester.ac.uk, richard.winpenny@manchester.ac.uk

Table of Contents

Experimental and Computational Details 4

Figure S1: MS data of **2**$^-$.......... 15

Figure S2: CID-MS spectra of **7**$^-$ (m/z = 4688) at E_{lab} = 160, 180 and 200 eV. 16

Figure S3: CID-MS spectra of **13**$^-$ (m/z = 2352) at E_{lab} = 55, 65, and 75 eV (a). 17

Figure S4: Normalized survival yield of **1**$^-$ vs E_{com} fitted to a sigmoidal Hill function. 18

Figure S5: Normalized survival yield of **3**$^-$ vs E_{com} fitted to a sigmoidal Hill function. 19

Figure S6: Normalized survival yield of **4**$^-$ vs E_{com} fitted to a sigmoidal Hill function. 20

Figure S7: Normalized survival yield of **5**$^-$ vs E_{com} fitted to a sigmoidal Hill function. 21

Figure S8: Normalized survival yield of **6**$^-$ vs E_{com} fitted to a sigmoidal Hill function. 22

Figure S9: Normalized survival yield of **8**$^-$ vs E_{com} fitted to a sigmoidal Hill function. 23

Figure S10: Normalized survival yield of **9**$^-$ vs E_{com} fitted to a sigmoidal Hill function. 24

Figure S11: Normalized survival yield of **10**$^-$ vs E_{com} fitted to a sigmoidal Hill function. 25

Figure S12: Normalized survival yield of **11**$^-$ vs E_{com} fitted to a sigmoidal Hill function. 26

Figure S13: Normalized survival yield of **13**$^-$ vs E_{com} fitted to a sigmoidal Hill function. 27

Table S1: Computational data of the carboxylic acids/carboxylates 28

Figure S14: Correlation between gas phase acidity of the carboxylates and the rings' E_{50} values. 29

Figure S15: Correlation between simulated pK_A value of the carboxylic acid and the rings' E_{50} values. 30

Figure S16: Correlation between the Weizsäcker kinetic energy of COO^- and the rings' E_{50} values. 31

Figure S17: Plot between the wavenumber of the asymmetric O-C-O stretching vibration and the rings' E_{50} values. 32

Figure S18: NCI plots of **7**$^-$. 33

Table S2. Crystallographic data for the compounds with **3**$^-$, **5**$^-$, **6**$^-$, **8**$^-$, **10**$^-$, **11**$^-$ and **13**$^-$. 35

Figure S19: Crystal structure of [$NH_3{}^n$Pr][**3**]. 38

Figure S20: Crystal structure of [NH_2(Allyl)$_2$][**5**]. 39

Figure S21: Crystal structure of [NH_2(Allyl)$_2$][**6**]. 40

Figure S22: Crystal structure of [$NH_2{}^n$Pr$_2$][**8**]. 41

Figure S23: Crystal structure of [$NH_2{}^n$Pr$_2$][**10**]. 42

Figure S24: Crystal structure of [$NH_2{}^n$Pr$_2$][**11**]. 43

Figure S25: Crystal structure of [$NH_2{}^n$Pr$_2$][**13**]. 44

References 45

Experimental and Computational Details

Synthesis, Materials and Sample Preparation

The complexes involving **1**$^-$,[1] **2**$^-$,[2] **4**$^-$,[3] **9**$^-$,[4] and **12**$^-$ [5] were synthesised according to previously published procedures. The amine cation was $[NH_2{}^nPr_2]^+$ (^{n}Pr = $^nC_3H_7$) in all five cases.

3$^-$**:** The amine cation was $[NH_3{}^nPr]^+$. $CrF_3 \cdot 4\ H_2O$ (6.0 g, 33 mmol), 3,3-dimethylbutyric acid (34 g, 293 mmol), propylamine (0.7 g, 12 mmol), and $2NiCO_3 \cdot 3\ Ni(OH)_2 . 4\ H_2O$ (0.8 g, 1.36 mmol) were heated at 160°C for 26 hours with stirring. The flask was left to cool to room temperature and acetone (80 mL) was added and stirred for 15 minutes. The solution was filtered and solvent was removed under a reduced pressure. Methanol (70 mL) was added to the resultant residue and stirred for 1 h. The solution was left to stand at room temperature for 48 h. The green crystalline product was filtered and washed with methanol (25 mL) and acetonitrile (50 mL), and dried under vacuum. **Yield:** 9.7 g (83 % calc. from $CrF_3 \cdot 4\ H_2O$ used). **Elemental analysis** (%) calcd. for $C_{99}H_{186}NiCr_7F_8NO_{32}$: C: 48.00, H: 7.57, N: 0.57, Ni: 2.37, Cr: 14.69; found: C: 48.17, H: 7.75, N: 0.63, Ni: 2.38, Cr: 13.92. Crystals were obtained from a solution of DCM/MeCN/MeOH. **MS** (nESI, negative): measured: 2416.6945, calculated: 2416.7239 (**3**$^-$).

5$^-$**:** The amine cation was $[NH_2(Allyl)_2]^+$. $CrF_3 \cdot 4\ H_2O$ (12.0 g, 66 mmol), 2-ethylbutyric acid (30 g, 258 mmol), diallylamine (2.4 g, 2.47 mmol), and $2\ NiCO_3 \cdot 3\ Ni(OH)_2 \cdot 4\ H_2O$ (2.0 g, 3.4 mmol) were heated at 140°C for 17 hours with stirring. The flask was then left to cool to room temperature and methanol (100 mL) was added and stirred for 4 hours. The product was filtered, washed with a large quantity of methanol (300 mL), dissolved in dichloromethane (200 mL), before the solution was filtered and solvent was removed under a reduced pressure. Acetone (300 mL) was added to the resultant residue and refluxed for 1 h before being left to stand at room temperature for 48 h. The green crystalline green product was filtered and washed with methanol (25 mL), and dried under vacuum. **Yield:** 16.0 g (67% calc. from $CrF_3 \cdot 4\ H_2O$ used). **Elemental analysis** (%) calcd. for $C_{102}H_{188}NiCr_7F_8NO_{32}$: C: 48.71, H: 7.53, N: 0.56, Ni: 2.33, Cr 14.47; found: C: 49.32, H: 7.68, N: 0.58, Ni: 2.37, Cr: 13.81. Crystals were obtained from a solution of acetone. **MS** (nESI, negative): measured: 2416.6986, calculated: 2416.7239 (**5**$^-$).

6⁻: The amine cation was $[NH_2(Allyl)_2]^+$. $CrF_3 \cdot 4\,H_2O$ (12.0 g, 66 mmol), 2-Methylbutyric acid (29 g, 284 mmol), diallylamine (2.4 g, 24.7 mmol), and $2\,NiCO_3 \cdot 3\,Ni(OH)_2 \cdot 4\,H_2O$ (2.0 g, 3.4 mmol) were heated at 140°C for 16 hours with stirring. The flask was then cooled to room temperature and acetonitrile (50 mL) was added and stirred for 2 hours. The product was filtered, washed with acetonitrile (150 mL), then dissolved in acetone (200 mL) and the solution was filtered, before the solvent was removed under a reduced pressure. The resultant residue was stirred with acetonitrile (100 mL) for 15 minutes, and the green crystalline product was filtered and dried under vacuum. **Yield:** 14.1 g (65%) calc. from $CrF_3 \cdot 4\,H_2O$ used). **Elemental analysis** (%) calcd. for $C_{86}H_{156}NiCr_7F_8NO_{32}$: C: 45.09, H: 6.86, N: 0.61, Ni: 2.56, Cr: 15.89; found: C: 45.24, H: 6.96, N: 0.65, Ni: 2.60, Cr: 15.32. Crystals were obtained from a solution of acetone/MeCN/MeOH. **MS** (nESI, negative): measured: 2192.4458, calculated: 2192.4729 (**6**$^-$).

7⁻: The amine cation was $[NH_2{}^nPr_2]^+$. Compound $[^nPr_2NH_2][Cr_7NiF_8(O_2CEt)_{16}]$ (3.0 g, 1.62 mmol),[5] 3,5-Bis(trifluoromethyl)benzoic acid (10.0 g, 38.74 mmol) and 1,2-dichlorobenzene anhydrous (5.0 ml) were stirred together in a Teflon flask at 160 °C for 19 hours then continued at 170 °C for 7 hours. The flask was allowed to cool to room temperature and acetonitrile was added (25 mL) and stirred for 30 min. The solid was filtered and washed with acetonitrile. The solid was then stirred with acetone (50 mL) for ca. 30 min and the obtained solution was filtered. The solvent from the filtrate was removed under reduced pressure leaving a green residue. **Yield:** 6.0 g (77%). **Elemental analysis** (%) calcd. for $C_{150}H_{64}NiCr_7F_{104}NO_{32}$: C: 37.61, H: 1.35, N: 0.29, Ni: 1.23, Cr: 7.60; found: C: 37.61, H: 1.12, N: 0.30, Ni: 1.29, Cr: 8.13. **MS** (nESI, negative): measured: 4687.5386, calculated: 4687.5708 (**7**$^-$).

8:⁻ The amine cation was $[NH_2{}^nPr_2]^+$. $[^nPr_2NH_2][Cr_7NiF_8(O_2CEt)_{16}]$ (2.5 g, 1.35 mmol),[5] 1-Naphthoic acid (10.0 g, 58.1 mmol) and 1,2-dichlorobenzene (5.0 ml) were stirred together in a Teflon flask at 160°C for 24 hours. The flask was allowed to cool to room temperature and acetone was added (30 mL) and stirred for 30 min. The solid was filtered and washed with acetone. The solid was then stirred with dichloromethane (150 mL) for *ca.* 30 min and the obtained solution was filtered and then placed on a silica column. Dichloromethane was used to elute first dense green band. Acetonitrile (100 mL) was added to the eluted solution (*ca.* 200 mL) and the solvent was slowly removed under reduced pressure to completely remove

dichloromethane and partially remove acetonitrile. The resultant microcrystalline product was collected by filtration, washed with acetone (25 mL) and dried under vacuum. **Yield:** 2.5 g (54%). **Elemental analysis** (%) calcd. for $C_{182}H_{128}NiCr_7F_8NO_{32}$: C: 64.00, H: 3.78, N: 0.41, Ni: 1.72, Cr: 10.66; found: C: 62.24, H: 3.50, N: 0.43, Ni: 1.61, Cr: 10.36. Crystals were obtained from a solution of DCM/MeCN. **MS** (nESI, negative): measured: 3313.1656, calculated: 3313.2258 (**8**$^-$).

10$^-$: The amine cation was $[NH_2{}^nPr_2]^+$. $CrF_3 \cdot 4\,H_2O$ (3.0 g, 16.57 mmol), 3,4,5-triethoxybenzoic acid (15 g, 3.17 mmol), dipropylamine (1.0 g, 9.88 mmol), and 2 $NiCO_3 \cdot 3\,Ni(OH)_2 \cdot 4\,H_2O$ (2.0 g, 3.4 mmol) were heated at 160 °C for 16 hours with stirring. The flask then cooled to room temperature and a mixture of acetonitrile with water 4:1 (200 mL) was added and stirred for 1 hour, before it was filtered, washed with a mixture of acetonitrile with water 4:1 (100 mL) and dried. The obtained powder was dissolved in diethyl ether (100 mL) and the solution was filtered through silica pad and diluted with heptane (100 mL). The solvents were slowly removed under reduced pressure to remove diethyl ether and partially heptane, and the solution was then left to stand at room temperature for 24 h. The green crystalline product was collected by filtration, washed with hexane (25 mL), and dried under vacuum. **Yield:** 4.7 g (42%) calc. from $CrF_3 \cdot 4\,H_2O$ used). **Elemental analysis** (%) calcd. for $C_{214}H_{288}NiCr_7F_8NO_{80}$: C: 54.35, H: 6.14, N: 0.30, Ni: 1.24, Cr: 7.70; found: C: 54.26, H: 6.31, N: 0.30, Ni: 1.22, Cr: 7.72. Crystals were obtained from a solution of diethylether/heptane. **MS** (nESI, negative): measured: 4626.1690, calculated: 4626.2354 (**10**$^-$).

11$^-$: The amine cation was $[NH_2{}^nPr_2]^+$. $CrF_3 \cdot 4H_2O$ (2.0 g, 11.04 mmol), *m*-toluic acid (10.0 g, 73.45 mmol), dipropylamine (0.73 g, 7.21 mmol), and 2 $NiCO_3 \cdot 3\,Ni(OH)_2 \cdot 4\,H_2O$ (0.5 g, 0.85 mmol) and 1,2-dichlorobenzene (8.0 ml) were stirred at 160°C for 24 hours. The flask was allowed to cool to room temperature and acetone was added (30 mL) stirred for 30 min. The solid was filtered and washed with acetone. After this, the solid was stirred with dichloromethane (100 mL) for *ca.* 30 min and obtained solution was filtered and diluted with acetone (100 mL). The dichloromethane was removed by distillation while stirring. The green microcrystalline product was collected by filtration, washed with acetone, and dried under vacuum. **Yield:** 2.8 g (62.5%) calc. from $CrF_3 \cdot 4\,H_2O$ used). **Elemental analysis** (%) calcd. for $C_{134}H_{128}NiCr_7F_8NO_{80}$: C: 56.69, H: 4.54, N: 0.49, Ni: 2.07, Cr: 12.82; found: C: 56.57, H: 4.34, N:

0.50, Ni: 2.12, Cr: 12.94. Crystals were obtained from a solution of DCM/toluene. **MS** (nESI, negative): measured: 2736.1728, calculated: 2736.2241 (**11**$^-$).

13$^-$**:** The amine cation was $[NH_2{}^nPr_2]^+$. $CrF_3 \cdot 4\,H_2O$ (5.0 g, 27.6 mmol), 2-furoic acid (25.0 g, 223 mmol), dipropylamine (1.5 g, 14.82 mmol), and 2 $NiCO_3 \cdot 3\,Ni(OH)_2 \cdot 4\,H_2O$ (0.8 g, 1.36 mmol) were stirred at 150 °C for five hours. The flask was allowed to cool to room temperature and acetone was added (30 mL) and stirred for *ca.* 30 min. The solid was filtered and washed with acetone. After this, the solid was stirred with dichloromethane (200 mL) for *ca.* 30 min and the obtained solution was filtered and diluted with acetone (70 mL). The dichloromethane was removed by distillation while stirring. The green microcrystalline product was collected by filtration, washed with acetone, and dried under vacuum. **Yield:** 4.3 g (44 %) calc. from $CrF_3 \cdot 4\,H_2O$ used). **Elemental analysis** (%) calcd. for $C_{86}H_{64}NiCr_7F_8NO_{48}$: C: 42.09, H: 2.63, N: 0.57, Ni: 2.39, Cr: 14.83; found: C: 41.42, H: 2.55, N: 0.55, Ni: 2.50, Cr: 14.27. Crystals were obtained from a solution of DCM/acetone. **MS** (nESI, negative): measured: 2351.6125, calculated: 2351.6404 (**13**$^-$).

All reagents and solvents were purchased from Sigma-Aldrich, Alfa, Fisher Scientific or Fluorochem and used without further purification. Syntheses of the complexes were carried out in Erlenmeyer Teflon FEP flasks supplied by Fisher. Final samples solutions were prepared between 150 and 200 µM in 4:1 toluene/methanol (**1**$^-$, **2**$^-$, **3**$^-$, **6**$^-$, **7**$^-$), THF (**2**$^-$, **3**$^-$, **4**$^-$, **5**$^-$, **6**$^-$, **7**$^-$) or 2:1 DCM/THF (**2**$^-$, **8**$^-$, **9**$^-$, **10**$^-$, **11**$^-$, **12**$^-$, **13**$^-$) respectively, depending on analyte solubility. Some compounds, in particular **2**$^-$, were tested under different conditions.

Crystallography

X-ray diffraction data for the compounds involving **3**$^-$ and **6**$^-$ were collected using a dual wavelength Rigaku FR-X rotating anode diffractometer using CuKα (λ = 1.54146 Å) radiation, equipped with an AFC-11 4-circle goniometer (κ and quarter-χ geometry, respectively), VariMAX^{TM} microfocus optics, a Hypix-6000HE detector and an Oxford Cryosystems plus nitrogen flow gas system (800 and 700, respectively), at a temperature of 100K. X-ray diffraction data for compounds involving **5**$^-$ and **11**$^-$ were collected using beamline i19 EH1 at Diamond Light source using Zr L-edge (λ = 0.6889 Å) radiation, equipped with a 3-circle Dual

Air-Bearing Fixed-χ Goniometer, a Pilatus 2M detector and an Oxford Cryosystems 800 plus nitrogen gas flow system, at a temperature of 100K.[6,7] X-ray diffraction data for compounds involving **8**$^{-}$ and **10**$^{-}$ were collected using a Bruker Prospector diffractometer, equipped with a 3-circle fixed-χ goniometer, a CuKα (λ = 1.54146 Å) Incoatec microfocus source, an Apex2 CCD detector and an Oxford Cryosystems Cryostream 700 nitrogen gas flow system, at a temperature of 150K. X-ray diffraction data for the complex with **13**$^{-}$ were collected using an Oxford Diffraction Xcalibur2 diffractometer, equipped with a 4-circle kappa goniometer, a MoKα (λ = 0.71073 Å) sealed tube source with a 0.5 mm collimator, a Sapphire2 CCD detector and an Oxford Instruments Cryojet nitrogen gas flow system, at a temperature of 100K. Data for the complexes with **3**$^{-}$, **6**$^{-}$, and **13**$^{-}$ were collected using Rigaku Oxford Diffraction CrysAlisPro.[8] Data for the complexes with **5**$^{-}$ and **11**$^{-}$ were collected using Generic Data Acquisition (GDA). Data for the compounds with **8**$^{-}$ and **10**$^{-}$ were collected using Bruker Apex2. All data were reduced using CrysAlisPro v43.[8] Absorption correction was performed using empirical methods (SCALE3 ABSPACK) based upon symmetry-equivalent reflections combined with measurements at different azimuthal angles.

The crystal structure was solved and refined against all F2 values using the SHELX and Olex2 suite of programmes.[9,10] Coordinates for all non-hydrogen atoms were freely refined and atomic displacement parameters were freely refined anisotropically. Hydrogen atoms were constrained to idealised positions with the coordinates refined to ride with the parent atom. All methyl groups were refined to have idealised staggered geometries, rather than allowing the torsion angles to be refined: allowing refinement of the torsion angles results in non-convergence of the model refinement. Hydrogen isotropic atomic displacement parameters were constrained to ride with the parent atom with an appropriate multiplier for the hybridisation.

In all cases, the carboxylate ligands were restrained to have similar 1,2- and 1,3- bond distances for similar moieties. This was applied to ensure good averaging across all similar moieties due to the large amounts of disorder evident in the electron density maps, or poor resolution of the datasets. Disordered components of the central threads were either restrained to have similar 1,2- and 1,3- distances, or fixed 1,2- and 1,3- distances, depending on the severity or close overlap of the disorder. The nickel sites were distributed evenly around the ring, with the metal site occupancies fixed such that each site averages to be

87.5% chromium and 12.5% nickel. In most cases, the {Cr_7Ni} rings are disordered over at least two positions in the unit cell. One of the issues with modelling this disorder is that, although some carboxylates are clearly disordered based on the direction of the long axis of the oxygen atomic displacement parameters, once one group is split, the entire structure must be split to refine the correct geometries around the metal sites. Therefore, modelling of this disorder can be limited by the observable diffraction limit for the samples.

Data for compound with **3**$^-$ were of sufficient resolution to refine the entire ring over two positions. Strong similar neighbour atomic displacement parameter and rigid bond restraints were applied globally to refine sensible atomic displacement parameters in light of the close overlap of the disordered atomic positions. The occupancies of the disordered parts were refined competitively against a single free variable to give relative occupancies of 0.506(5) and 0.494(5), respectively, approximating to a 50% disorder of the ring. The central thread did not refine to the same occupancies, and the two positions were refined competitively against a free variable to give refined occupancies of 0.751(15) and 0.249(15), respectively. Near the primary ammonium cation, there are three positions (O33, O34 and O35) that correspond to a disordered water molecule. The occupancies for these positions were refined such that each water was designated an independent free variable and the sum of those free variables was fixed at 100% occupied. Although refined independently, the occupancy for O34 is 0.246(4), which is the same as the minor disordered part of the ammonium cation thread within error, which would indicate that this disorder is driven by the thread-solvent interactions, rather than through interactions with the ring.

The compound with **5**$^-$ contains two independent molecules in the asymmetric unit. Disorder was refined over two positions where required, with the occupancy refined competitively against individual free variables for each disordered moiety.

Data for Compound **6** were of a resolution below the recommended IUCr minimum. The molecule exhibits large apparent thermal motion, likely due to closely overlapping disorder of the entire structure. However, the data resolution is too low to be able to sufficiently model this disorder and maintain a reasonable data to parameter ratio. Where there is clear disorder of the isobutyl groups and central diallyl thread, these have been refined over two positions,

with the occupancy refined competitively against individual free variables for each disordered moiety.

Data for Compound **8** were of a resolution well below the recommended IUCr minimum. The symmetry of the system is such that only half the ring is present in the asymmetric unit. The ring is likely disordered with very close overlap over two disordered positions. However, the data resolution is too low to be able to sufficiently model this disorder and maintain a reasonable data to parameter ratio. The central thread was found to be disordered across the 2-fold rotation symmetry operation at the centre of the ring. Therefore, this was refined as a 50% occupancy whole molecule over the special position set with a non-interacting with symmetry generated equivalents part code. Where there is clear disorder of the napthalene groups, these have been refined over two positions, with the occupancy refined competitively against individual free variables for each disordered moiety. Solvent molecules were refined with isotropic displacement parameters to reduce the number of refinable parameters considering the limited number of data points.

Data for the compound with **10**$^-$ were of a resolution well below the recommended IUCr minimum. The symmetry of the system is such that only a quarter of the ring is present in the asymmetric unit. Although the central ring does not show signs of total ring disorder, the 2,3,4-trimethoxybenzyl moieties do exhibit very large atomic displacement parameters, indicative of extensive disorder of these groups. This has been modelled over two positions where possible, given the limited data set, with the occupancy refined competitively against individual free variables for each disordered moiety. The central thread was also found to be disordered about the 4-fold rotation axis through the centre of and perpendicular to the plane of the ring. This was modelled as a whole molecule over the special position set with a non-interacting with symmetry generated equivalents part code with the occupancy fixed at 25%.

Data for the compound involving **11**$^-$ were of sufficient resolution to refine the entire ring over two positions. Similar neighbour atomic displacement parameter and rigid bond restraints were applied globally to refine sensible atomic displacement parameters considering the close overlap of the disordered atomic positions. The occupancies of the disordered parts were initially refined competitively against a single free variable to give

relative occupancies of 0.502(6) and 0.498(6), respectively, approximating to a 50% disorder of the ring. This was subsequently fixed at 50% occupancy. The axial carboxylates exhibit much larger atomic displacement parameters than the equatorial counterparts, which is in indicator of a much greater degree of disorder of these groups. However, this disorder could not be further modelled due to the poor data to parameter ratio this would have introduced.

Data for the compound with **13**$^-$ were of sufficient resolution to refine the entire ring over two positions. However, any disorder of the central ring is so closely overlapped that it was not possible to create and refine a stable model to account for this. Furan moieties were modelled over two positions where required, with the occupancy refined competitively against individual free variables for each disordered moiety. The central thread was found to be disordered over two positions (four when symmetry operations are taken into account) and so this was modelled over two positions, with the occupancy refined competitively against a single free variables to 50% occupied.

Collision-Induced Dissociation Mass Spectrometry (CID-MS) and Data Processing

Samples were ionised and transferred to the gas phase with a nESI source and were sprayed from borosilicate glass capillaries (World Precision Instruments, Stevenage, UK). The latter were pulled on the Flaming/Brown P-2000 laser puller (Sutter Instrument Company, Novato, CA, US). The capillary voltage (typically 1.3 - 4 kV) was applied through a platinum wire (Diameter 0.125 mm, Goodfellow, Huntingdon, UK) inserted into the nESI capillaries. Source temperatures between 30 °C and 200 °C were applied.

The Q Exactive Ultra-High-Mass-Range (UHMR) Hybrid Quadrupole-Orbitrap Mass Spectrometer (Thermo Fisher) was used for the derivation of the E_{50} values *via* CID-MS experiments.[11] The ring anions **1**$^-$ – **13**$^-$ were obtained directly from sample solution, or their signal was further enhanced in some cases with in-source trapping at 200 V. Target ions were *m/z*-isolated in a quadrupole filter, accelerated to a user-defined kinetic energy (E_{lab}: 0 – 300 eV) and injected into the higher-energy C-trap dissociation (HCD) cell, which contained nitrogen gas (trapping gas pressure parameter: 2.0). Non-fragmented precursor ions and fragment ions were transferred to the Orbitrap mass analyser (AGC target: 3E6 ions, maximum inject time: 100 ms, resolution: 25000).

E_{50} values were obtained as described elsewhere.[12–15] Mass spectra were obtained at different collision energies, and the ratio of the precursor ion intensity, relative to the total ion intensity ('survival yield', *SY*), was plotted vs the collision energy in the centre-of-mass frame (E_{com}, Figure 2b). *SY* plots were fitted with a sigmoidal Hill function (Hill1 function in OriginPro 2020b), yielding the transition point (E_{50}) at which *SY* reaches 0.5 or 50%.

Density Functional Theory (DFT)

All quantum chemical calculations were performed with the ORCA 6.0.0 and 6.0.1 RELEASE version on the Computational Shared Facility at The University of Manchester.[16,17] The wavefunctions were analysed with Multiwfn Version 3.8(dev)[18] for non-covalent interactions.[19]

The carboxylate and carboxylic acid structures were optimised using the ωB97M-D4rev[20–22] functional in combination with the def2-TZVPD basis set.[23,24] Frequency calculations were performed within the Quasi Rigid Rotor Harmonic Oscillator (QRRHO) approximation[25] using the same functional/basis set combination (T = 298.15 K; ideal gas p = 1 bar). The absence of imaginary frequencies confirmed that the obtained geometries were local minima. For the simulations of solution phase properties (pK_a), the solvation model based on solute density (SMD) with default settings for water was additionally employed to the above methods.[26] Verytight convergence criteria were set for the geometry optimisation and SCF convergence throughout.

Additionally, DLPNO-CCSD(T)[27] single point energies with a ANO-pVQZ[28] basis set were obtained for verifying the trend of the proton affinities obtained with above density functional. Verytight and tightpno convergence criteria and the RIJCOSX[29] approximation in combination with the AutoAux[30] keyword were used.

The polymetallic ring anion **7**⁻ was optimised with the r2scan-3c[31] composite method in the gas phase using normal convergence criteria (!OPT). A multiplicity of 24 was set for all complexes. No frequency calculations were performed due to their high computational demand. Single point calculations with the ωB97M-V[21,32] functional and def2-SVPD[24] basis set using the self-consistent treatment of the dispersion correction (!SCNL) and verytight SCF

convergence criteria (!verytightscf) were performed for subsequent analysis of the wavefunction for noncovalent interactions with Multiwfn.

Calculation of Proton Affinity

The proton affinity (PA) is the negative enthalpy change of the gas phase reaction between a proton and a second reactant, yielding the conjugate acid of that reactant.[33]

$$RCO_{2(g)}^{-} + H_{(g)}^{+} \rightarrow RCO_2H_{(g)}$$

$$PA = -[H(RCO_2^-) + H(H^+) - H(RCO_2H)]$$

The PA was calculated accordingly, estimating the proton enthalpy as $\frac{5}{2}RT$. The electronic energy and the thermostatistical corrections E_{cor} to the electronic energy for obtaining the enthalpy were derived from the gas phase frequency calculation. The inner energy *U* is the sum of the electronic energy E_{el}, the zero point vibrational energy E_{ZPE}, the finite temperature correction to E_{ZPE} E_{vib}, and the rotational and translational thermal energies E_{rot} and $E_{vib.}$

$$U = E_{el} + E_{ZPE} + E_{vib} + E_{rot} + E_{trans}$$

$$E_{cor} = E_{ZPE} + E_{vib} + E_{rot} + E_{trans}$$

$$U = E_{el} + E_{cor}$$

The enthalpy *H* is obtained from the inner energy *U*

$$H = U + k_B \cdot T$$

Where k_B is the Boltzmann constant and T the temperature.

The correlation in Figure 4 shows only the enthalpic correction term E_{cor}, which correlates better with the E_{50} value than considering the enthalpy with the electronic term.

Calculation of pK_A values

The pK_a values of the carboxylic acids were calculated based on an isodesmic reaction between the respective acid RCO_2H and a reference carboxylic acid BH.[34] Here, we chose benzoic acid as the reference acid with a tabulated pK_a of 4.2.[35]

$$RCO_2H_{(aq)} + B^-_{(aq)} \rightarrow RCO^-_{2(aq)} + BH_{(aq)}$$

The equilibrium constant *K* for this reaction can be expressed in the form of the dissociation constants K_a for RCO_2H and BH

$$K_{a,RCO_2H} = \frac{\left[RCO_2^-\right]\left[H_3O^+\right]}{\left[RCO_2H\right]}, \qquad K_{a,BH} = \frac{\left[B^-\right]\left[H_3O^+\right]}{[BH]}$$

$$K = \frac{\left[RCO_2^-\right][BH]}{\left[RCO_2H\right]\left[B^-\right]} = K_{a,RCO_2H}) - log(K_{a,BH})$$

This expression allows for the calculation of pK_{a,RCO_2H} from the reaction free energy and the experimental pK_a value of BH

$$\Delta G = G(BH) + G\left(RCO_2^-\right) - G\left(RCO_2H\right) - G\left(B^-\right)$$

$$\Delta G = - RTK)$$

$$pK_{a,RCO_2H} = \frac{\Delta G}{2.303RT} + pK_{a,BH}$$

Weizsäcker kinetic energy

The Weizsäcker kinetic energy (T_W) of the carboxylate group were calculated with Multiwfn. The fragments were portioned based on atoms in molecules basin analysis, and the generated basins were integrated using the Weizsäcker functional (isuerfunc=40).

The Weizsäcker kinetic energy is a electron density functional, and is expressed as

$$T_W = \frac{|\nabla\rho(r)|^2}{8\rho(r)}$$

where $\nabla\rho(r)$ is the gradient of the electron density ρ at point r. In this work, T_W is used as a proxy for the electron density of the carboxylate fragment and the residue fragment.

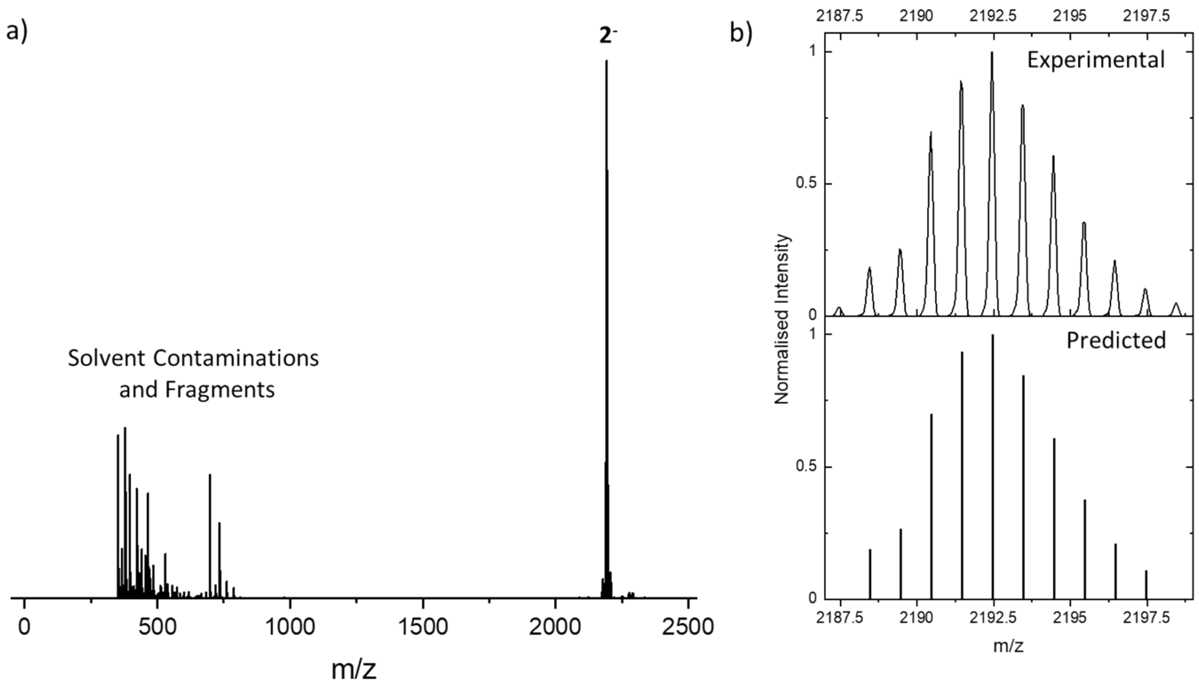

Figure S1: MS data of **2**$^-$. a) Mass spectrum of [$NH_2{}^nPr_2$][**2**] in negative ion mode. The **2**$^-$ ion was found as the main peak. b) Comparison between predicted and experimental isotopic distribution of **2**$^-$.

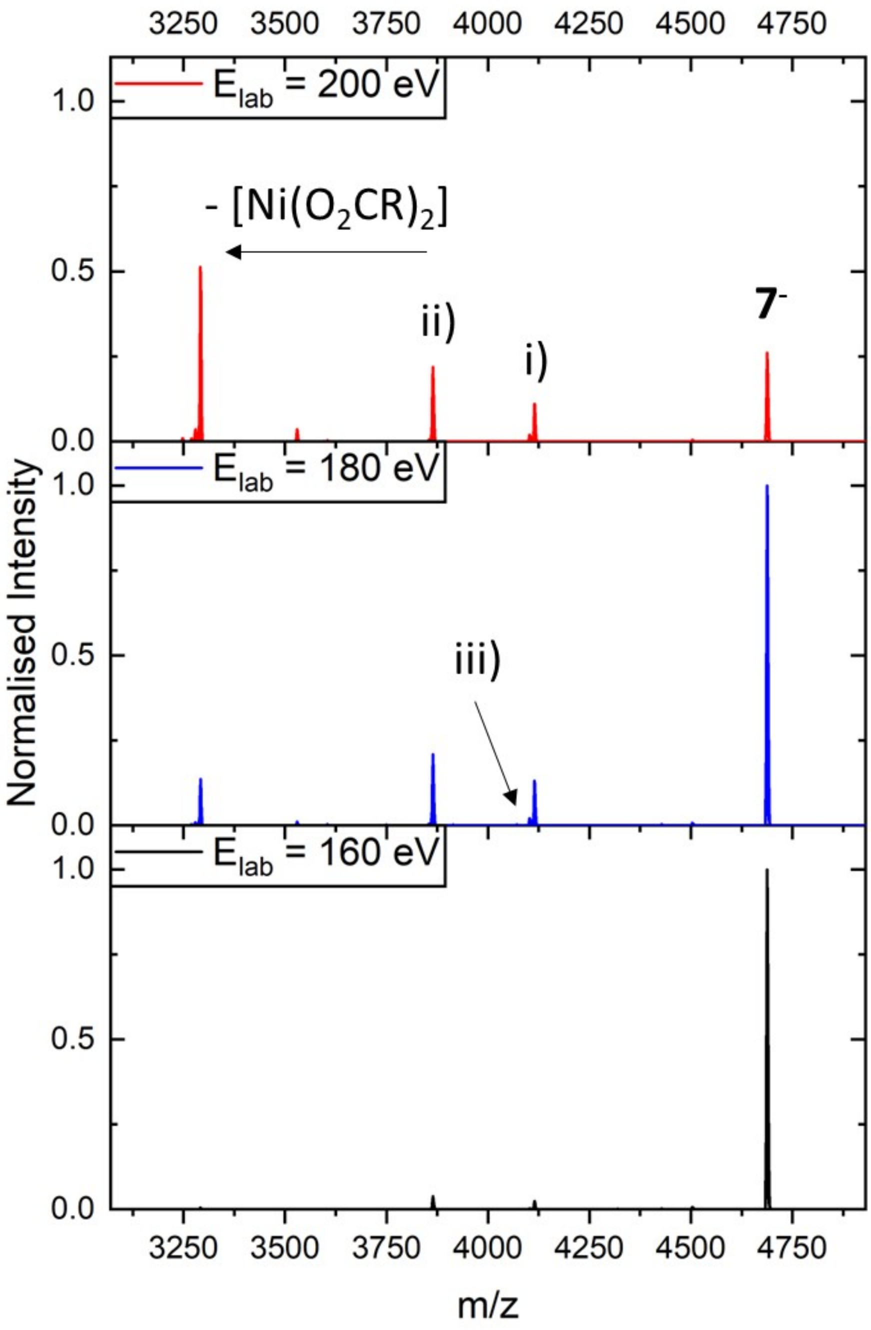

Figure S2: CID-MS spectra of **7**⁻ (m/z = 4688) at E_{lab} = 160, 180 and 200 eV. The dominant primary fragmentation channel involves the loss of [Cr(O_2CR)$_3$] to fragment ii), conversely to the behaviour other ring anions as discussed in the main text and illustrated in Figure 2a. Further fragmentation follows similar channels, as shown with the example of [Ni(O_2CR)$_2$] loss from ii).

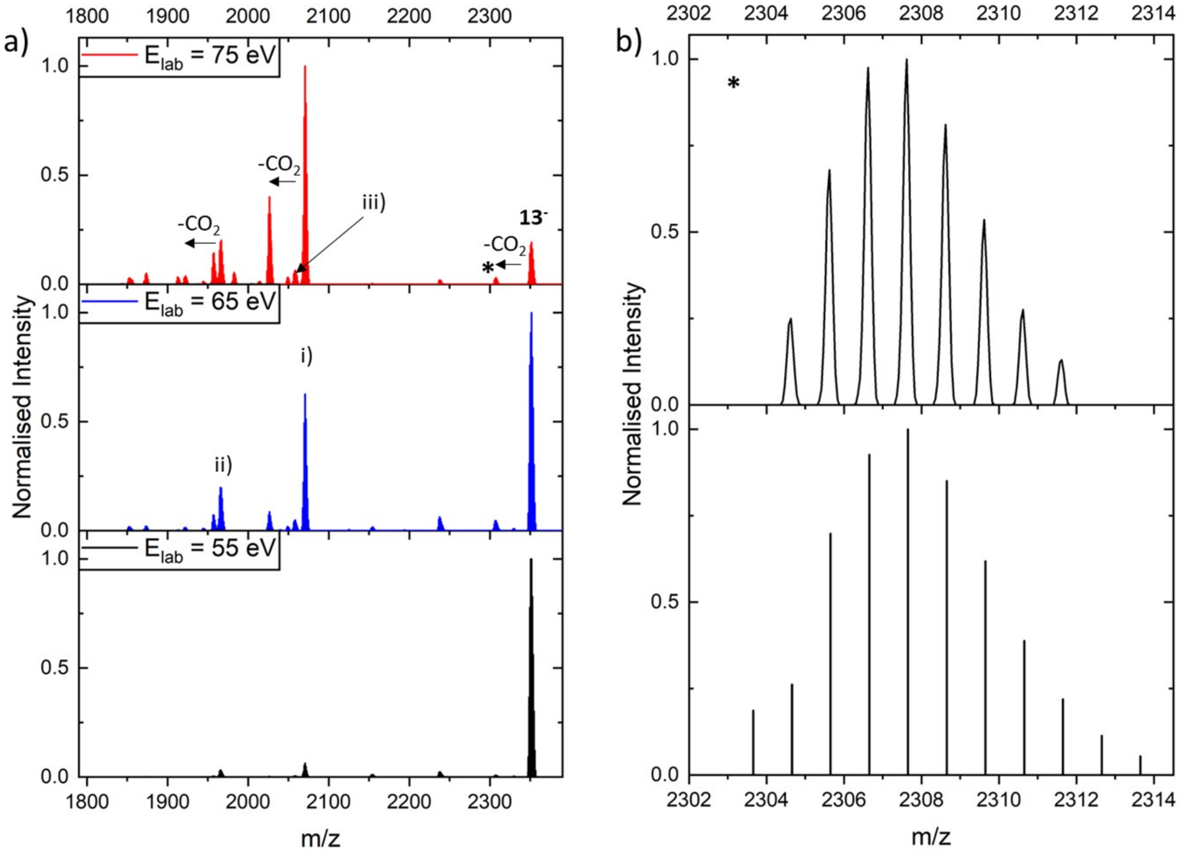

Figure S3: CID-MS spectra of **13**$^-$ (m/z = 2352) at E_{lab} = 55, 65, and 75 eV (a). The fragmentation pattern is more diverse than those of the other ring anions (Figure 2a, only main fragments are i, ii and iii), involving the loss of CO_2 from the precursor and the main fragment ions. The difference in fragmentation behaviour compared to the other ions could be related to interactions between the metal centres and the furan oxygen, possibly leading to bond breaking of the M-O bonds to the carboxylic group and the release of CO_2. These channels are however not dominant. b) Comparison between measured (top) and predicted (bottom) isotopic pattern of [**13**$^-$ – CO_2]$^-$ (m/z = 2307). Slight deviations are observed due to MS-MS selection, however the agreement in accurate mass is excellent.

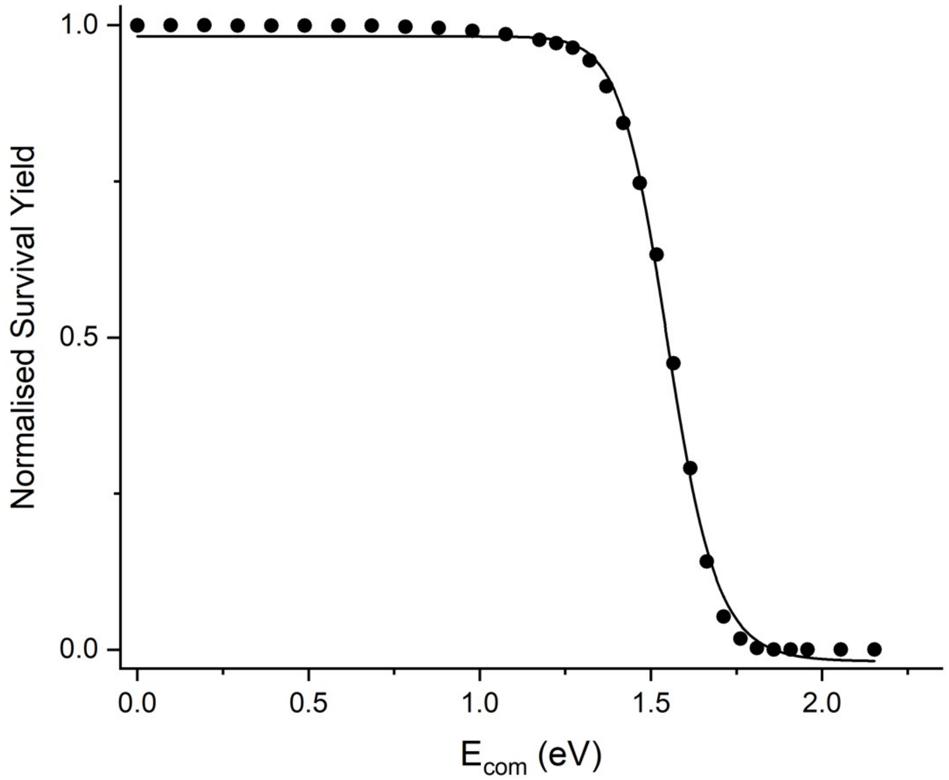

Figure S4: Normalized survival yield of **1**$^{-}$ vs E_{com} fitted to a sigmoidal Hill function. An E_{50} value of (1.552 ± 0.023) eV was determined.

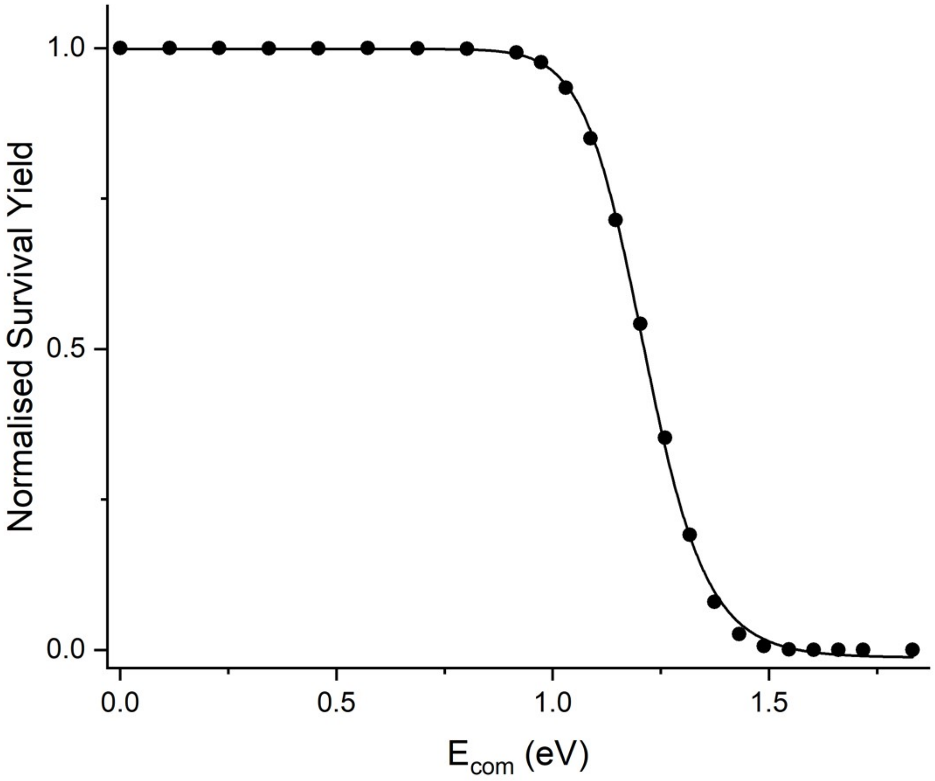

Figure S5: Normalized survival yield of **3**$^{-}$ vs E_{com} fitted to a sigmoidal Hill function. An E_{50} value of (1.207 ± 0.018) eV was determined.

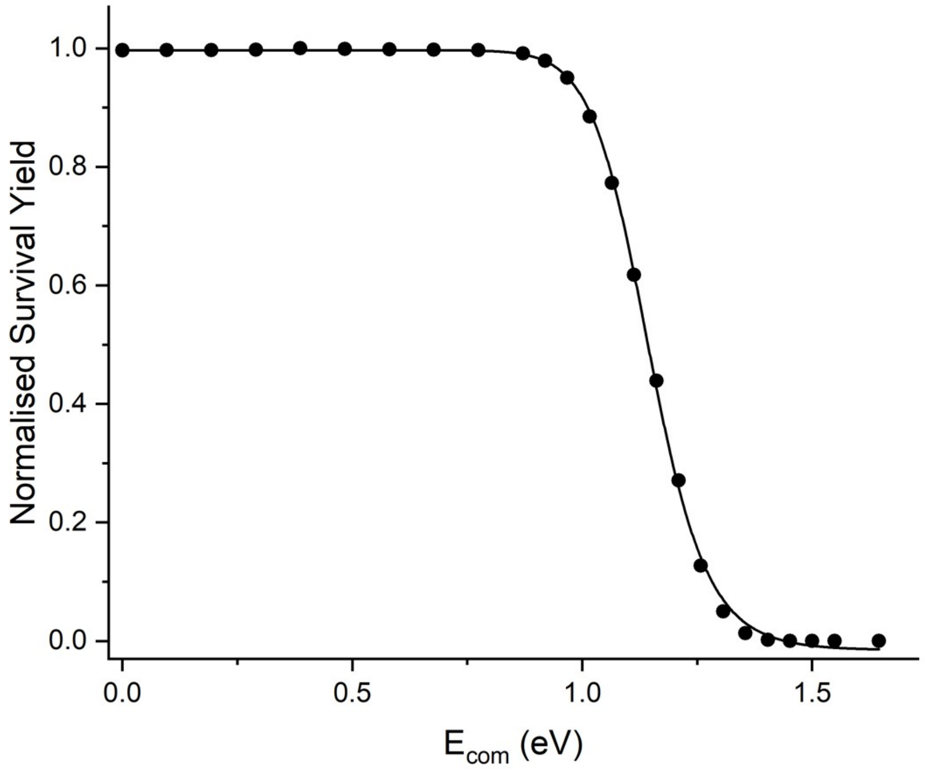

Figure S6: Normalized survival yield of **4**$^-$ vs E_{com} fitted to a sigmoidal Hill function. An E_{50} value of (1.145 ± 0.017) eV was determined.

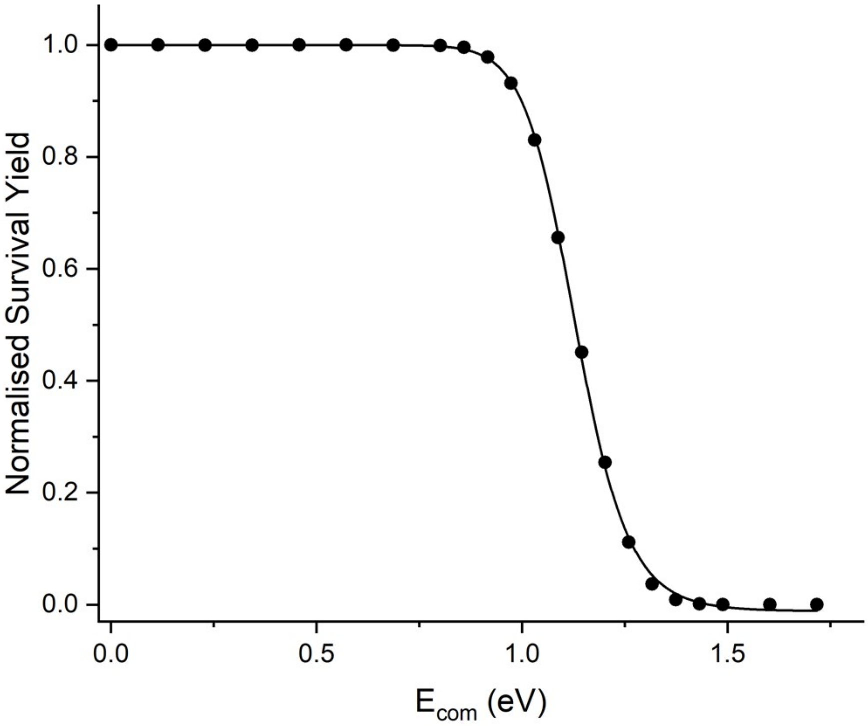

Figure S7: Normalized survival yield of **5**$^-$ vs E_{com} fitted to a sigmoidal Hill function. An E_{50} value of (1.131 ± 0.017) eV was determined.

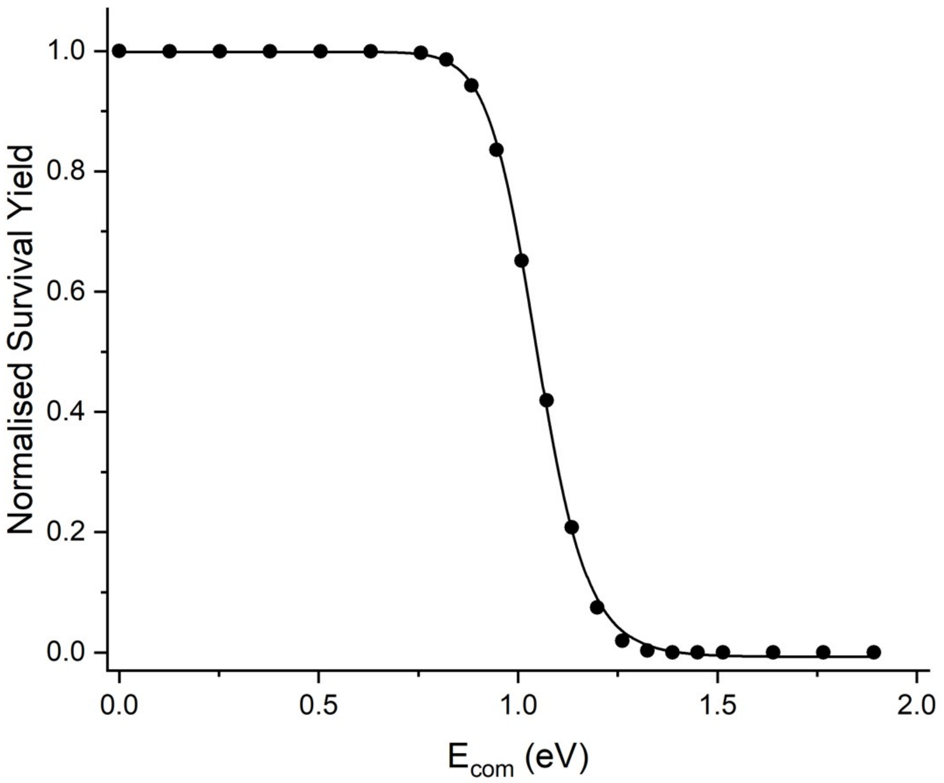

Figure S8: Normalized survival yield of **6**$^-$ vs E_{com} fitted to a sigmoidal Hill function. An E_{50} value of (1.047 ± 0.016) eV was determined.

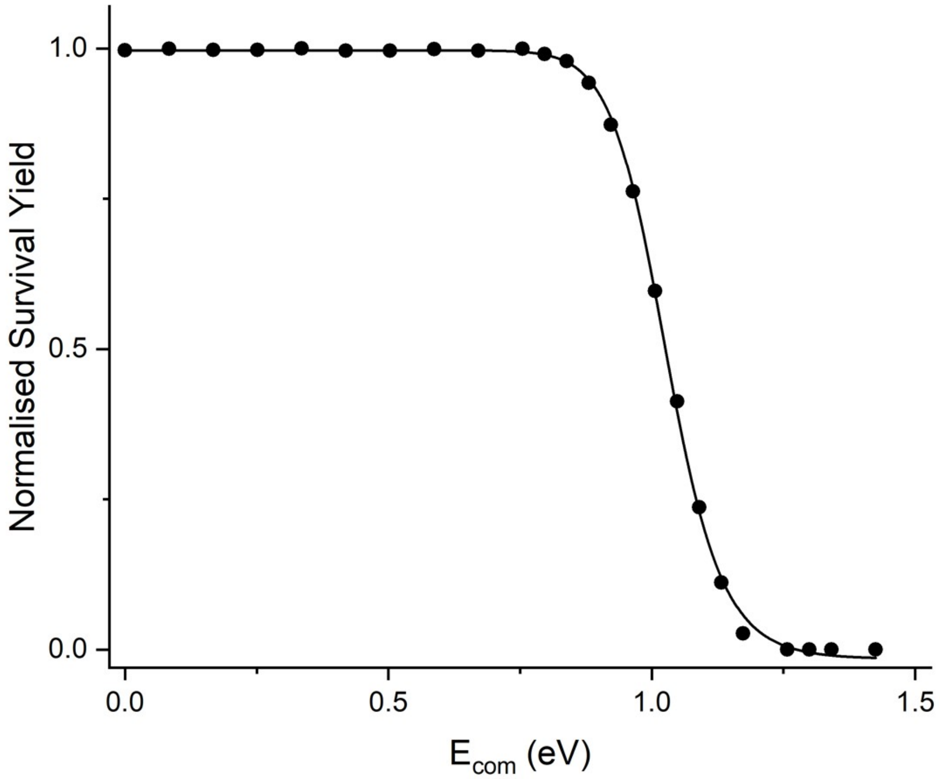

Figure S9: Normalized survival yield of **8**$^-$ vs E_{com} fitted to a sigmoidal Hill function. An E_{50} value of (1.028 ± 0.015) eV was determined.

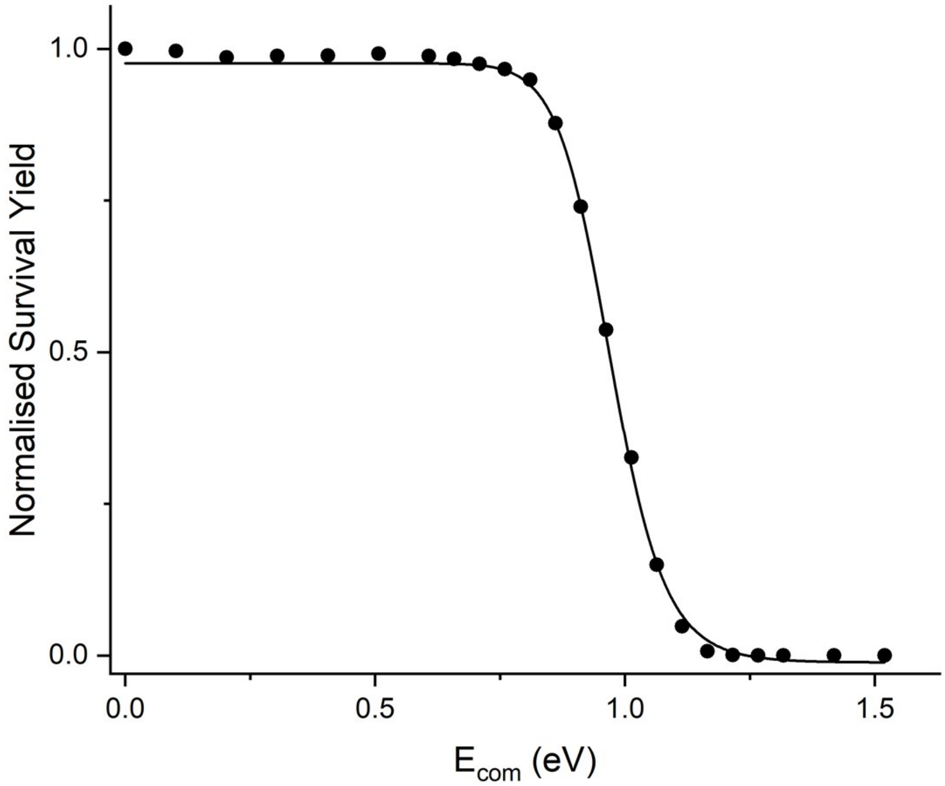

Figure S10: Normalized survival yield of **9**$^{-}$ vs E_{com} fitted to a sigmoidal Hill function. An E_{50} value of (0.972 ± 0.015) eV was determined.

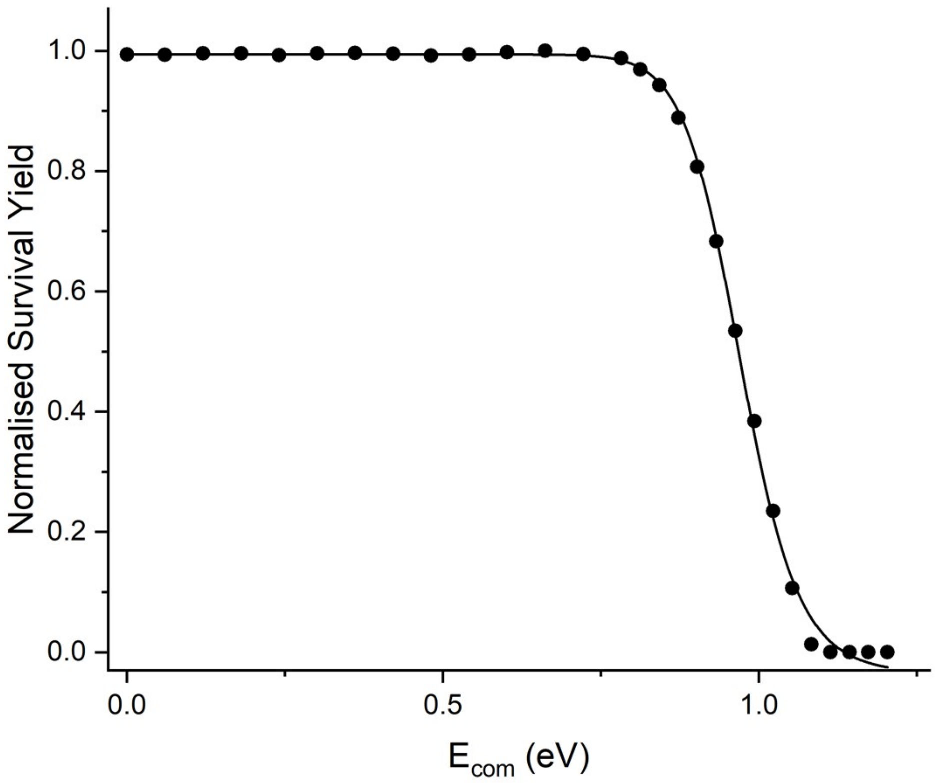

Figure S11: Normalized survival yield of **10**$^{-}$ vs E_{com} fitted to a sigmoidal Hill function. An E_{50} value of (0.971 ± 0.015) eV was determined.

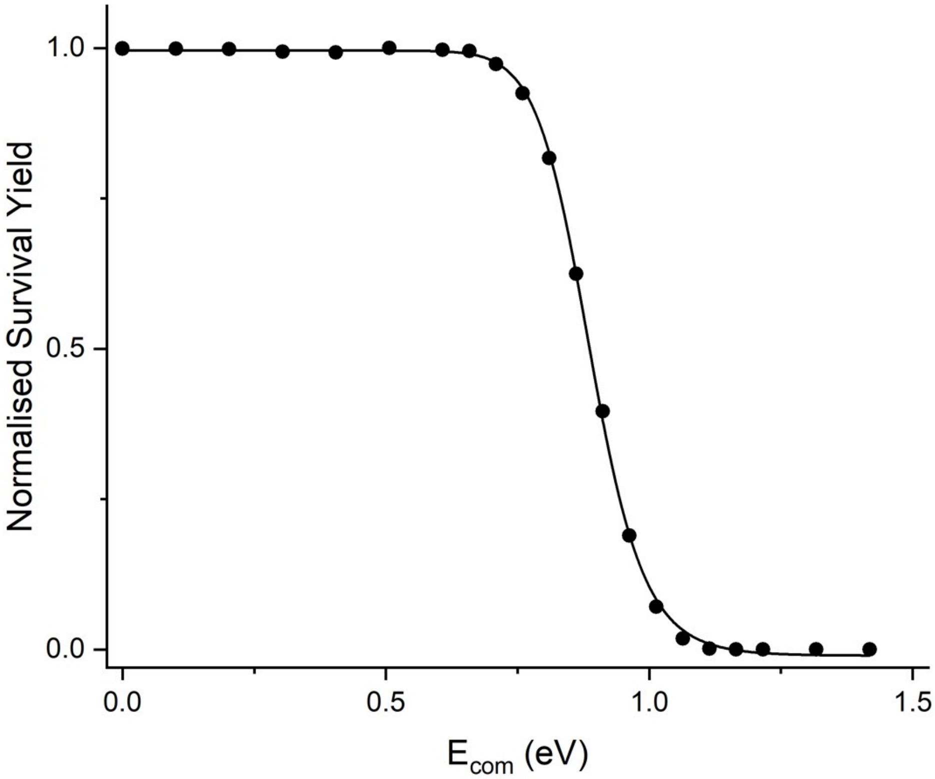

Figure S12: Normalized survival yield of **11**$^{-}$ vs E_{com} fitted to a sigmoidal Hill function. An E_{50} value of (0.888 ± 0.013) eV was determined.

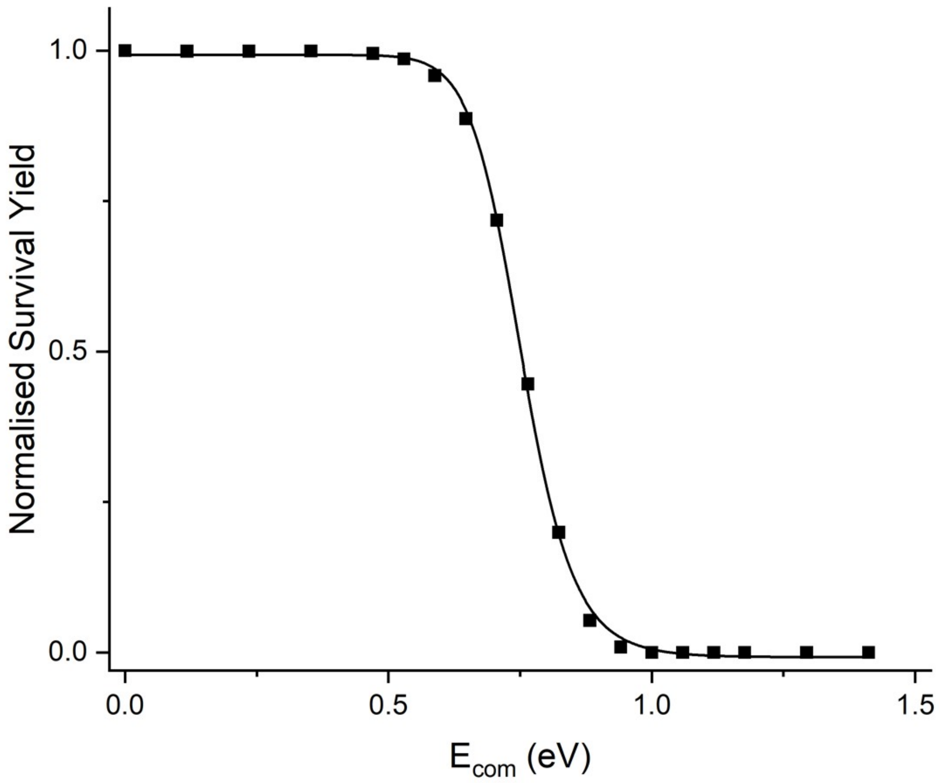

Figure S13: Normalized survival yield of **13**$^{-}$ vs E_{com} fitted to a sigmoidal Hill function. An E_{50} value of (0.753 ± 0.011) eV was determined.

Ring	Proton Affinity of $RCOO^-$ (kcal/mol) ωB97M-D4rev	Proton Affinity of $RCOO^-$ (kcal/mol) DLPNO-CCSD(T)	ΔE_{cor} (kcal/mol)	pK_A Value of RCOOH simulated	Weizsäcker Kinetic Energy of COO^- (a.u.)	O-C-O Asymmetric Stretching Vibration Wavenumber (cm^{-1})
**1^-**	339.69	344.91	9.07	5.01	143.928	1826.37
**2^-**	341.75	346.93	8.97	5.17	143.959	1831.22
**3^-**	341.83	346.96	8.94	4.95	143.972	1832.37
**4^-**	340.23	345.99	8.92	4.95	143.957	1830.88
**5^-**	341.39	346.43	8.97	4.84	143.957	1831.17
**6^-**	342.22	347.34	8.92	4.96	143.973	1833.96
**7^-**	324.14	328.44	8.66	1.78	143.984	1833.98
**8^-**	335.17	339.79	8.85	2.68	143.961	1812.74
**9^-**	337.99	342.58	8.82	3.36	143.960	1814.78
**10^-**	336.50	339.38	8.74	3.41	143.979	1815.73
**11^-**	338.11	342.80	8.73	3.83	143.983	1821.25
**12^-**	337.54	342.18	8.71	3.45	143.983	1822.26
**13^-**	336.71	341.27	8.62	2.12	144.000	1838.57

Table S1: Computational data of the carboxylic acids/carboxylates used in the respective ring anions **1**$^-$ – **13**$^-$.

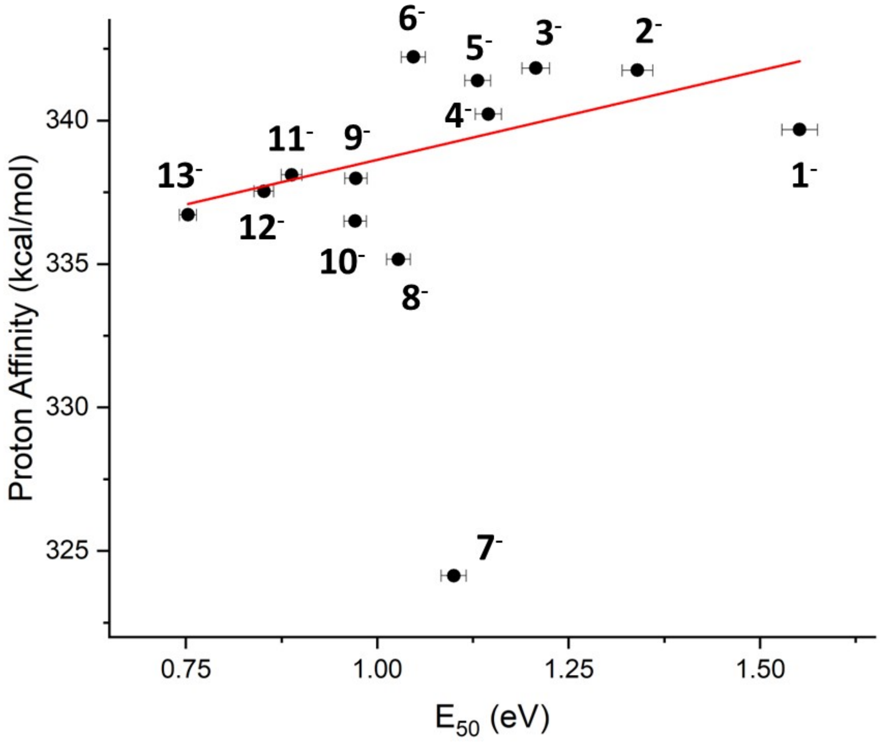

Figure S14: Correlation between gas phase acidity of the carboxylates and the rings' E_{50} values. R^2 = 0.33 when excluding the outlier **7**$^-$. Apart from **7**$^-$, the aliphatic (**1**$^-$ – **6**$^-$) and aromatic carboxylates (**8**$^-$ – **13**$^-$) and their respective rings group in two different regions. This trend above (ωB97M-D4rev) is highly similar to the one obtained with DLPNO-CCSD(T) (Table S1), suggesting that the functional has only little influence on the correlation with proton affinity.

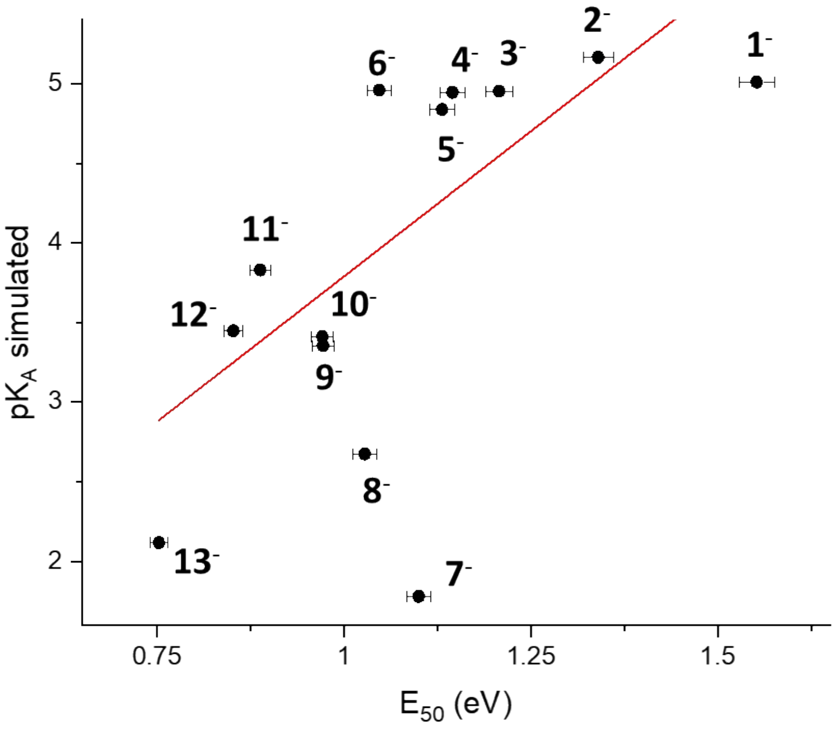

Figure S15: Correlation between simulated pK_A value of the carboxylic acid and the rings' E_{50} values. R^2 = 0.59 when excluding the outlier **7**$^-$. Experimental pK_A values were not found consistently for all carboxylic acids, and the pK_A values were therefore simulated.

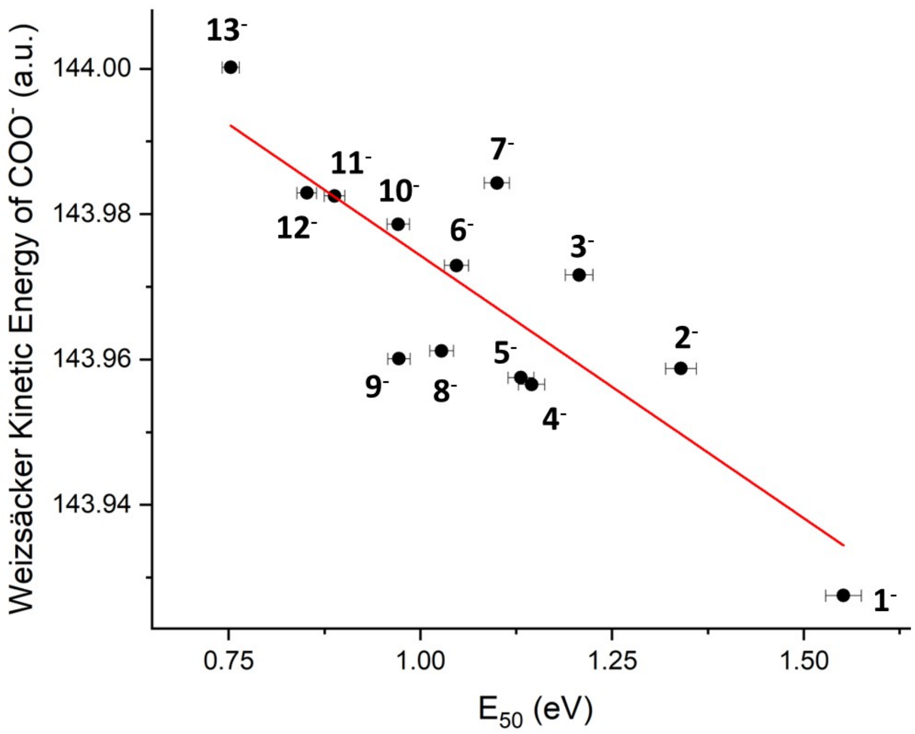

Figure S16: Correlation between the Weizsäcker kinetic energy of COO^- and the rings' E_{50} values. R^2 = 0.71. The Weizsäcker kinetic energy is a proxy for electron density, and lower electron density in the COO^- group will result in weaker bonds to electrophiles such as Ni^{II} and Cr^{III}.

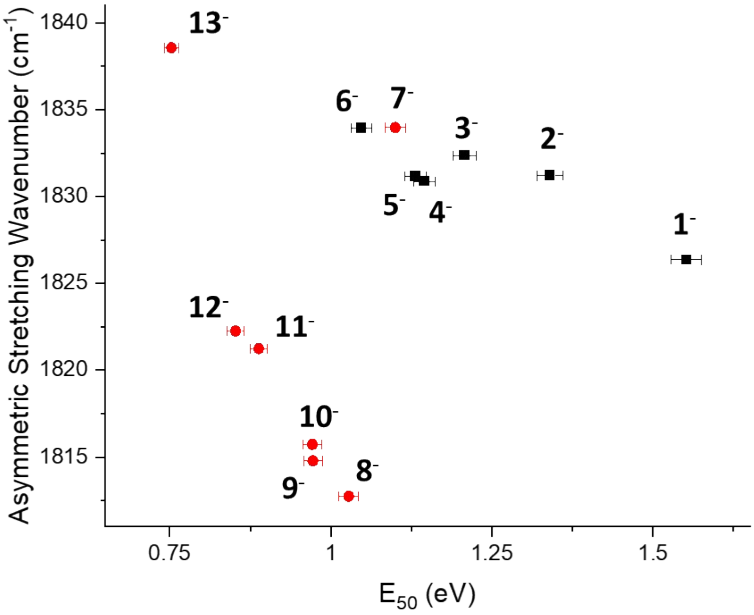

Figure S17: Plot between the wavenumber of the asymmetric O-C-O stretching vibration and the rings' E_{50} values. Apart from the outlier **7**⁻, different trends were found for aliphatic (black) and aromatic (red) carboxylates.

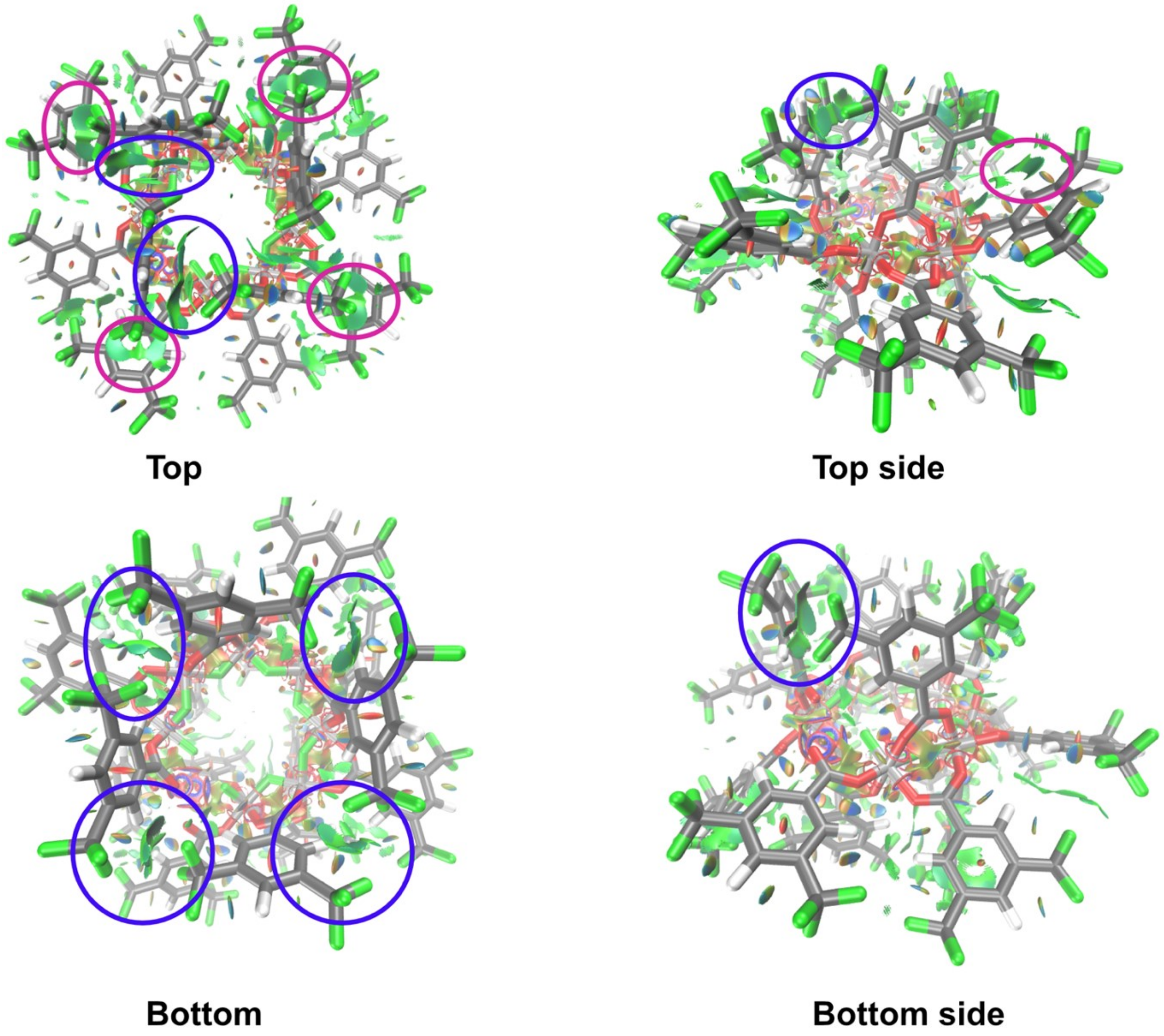

Figure S18: NCI plots of **7**$^{-}$. Blue circles indicate regions of axial carboxylate ligands interacting, purple indicates interactions between axial and equatorial carboxylate ligands. Plots were created for RDG isosurface of 0.55, with blue-green-red scale according to values of sign(λ_2)ρ, ranging from −0.02 to 0.025 au.

The isosurfaces show extensive weak interaction between i) axial-axial carboxylate ligands, ii) axial-equatorial carboxylate ligands. In the optimised structure the equatorial ligands moved away from the equatorial plane towards one side of the axis of the complex and their electron deficient π-system engages in F⋯π interaction with the CF_3 groups of the axial positioned carboxylate ligands (top and top side). Thus, this interaction between axial and equatorial ligands is restricted to one end of the complex.

The axial-axial interaction occurs at both ends of the complex (blue highlights). At the top of the axis, this interaction is manifested as a F$\cdots\pi$ interaction (top and top side). On the opposite side, an interaction of the CF_3 groups with an oppositely positioned CF_3 carbon atom leading to an interaction that might be interpreted as a $F_2CF\cdots CF_3$ tetrel bond is the observed pattern.

Table S2. Crystallographic data for the compounds with **3**$^-$, **5**$^-$, **6**$^-$, **8**$^-$, **10**$^-$, **11**$^-$ and **13**$^-$.

Code	$[NH_3{}^nPr]$[**3**]	$[NH_2(Allyl)_2]$[**5**]	$[NH_2(Allyl)_2]$[**6**]
Empirical formula	$C_{101}H_{191.49}Cr_7F_8N_2NiO_{33.24}$	$C_{102}H_{188}Cr_7F_8NNiO_{32}$	$C_{86}H_{160}Cr_7F_8NNiO_{32}$
Formula weight	2540.67	2515.23	2294.85
Temperature/K	100.00(10)	100.00(10)	99.9(4)
Crystal system	orthorhombic	triclinic	monoclinic
Space group	$P2_12_12_1$	P-1	$P2_1/c$
a/Å	19.4235(2)	19.0912(4)	19.1697(5)
b/Å	21.7382(2)	22.0288(4)	29.3283(8)
c/Å	31.1018(3)	30.5452(5)	20.8382(7)
α/°	90	90.1198(15)	90
β/°	90	90.1205(15)	104.138(3)
γ/°	90	106.2775(17)	90
Volume/Å^3	13132.2(2)	12331.0(4)	11360.7(6)
Z	4	4	4
ρ_{calc}g/cm^3	1.285	1.355	1.342
μ/mm^{-1}	5.407	0.760	6.182
F(000)	5382.0	5324.0	4828.0
Crystal size/mm^3	0.687 × 0.525 × 0.153	0.357 × 0.186 × 0.157	0.108 × 0.087 × 0.048
Radiation	Cu Kα (λ = 1.54184)	synchrotron (λ = 0.6889)	Cu Kα (λ = 1.54184)
2Θ range for data collection/°	4.96 to 155.774	3.184 to 51.006	4.754 to 128.144
Index ranges	-23 ≤ h ≤ 24, -19 ≤ k ≤ 27, -39 ≤ l ≤ 38	-23 ≤ h ≤ 23, -27 ≤ k ≤ 27, -38 ≤ l ≤ 38	-22 ≤ h ≤ 20, -33 ≤ k ≤ 32, -24 ≤ l ≤ 23
Reflections collected	114583	164196	54250
Independent reflections	27486 [R_{int} = 0.0602, R_{sigma} = 0.0462]	49932 [R_{int} = 0.0743, R_{sigma} = 0.0743]	18420 [R_{int} = 0.0441, R_{sigma} = 0.0544]
Data/restraints/parameters	27486/17182/2734	49932/17521/3097	18420/7908/1786
Goodness-of-fit on F^2	1.012	1.036	1.058
Final R indexes [I>=2σ (I)]	R_1 = 0.0584, wR_2 = 0.1614	R_1 = 0.1397, wR_2 = 0.3853	R_1 = 0.0902, wR_2 = 0.2708
Final R indexes [all data]	R_1 = 0.0664, wR_2 = 0.1690	R_1 = 0.1679, wR_2 = 0.4002	R_1 = 0.1629, wR_2 = 0.3357
Largest diff. peak/hole / e Å^{-3}	0.81/-0.45	1.20/-1.27	0.93/-0.61
Flack parameter	0.003(3)		

Code	$[NH_2{}^nPr_2]$[**8**]	$[NH_2{}^nPr_2]$[**10**]	$[NH_2{}^nPr_2]$[**11**]
Empirical formula	$C_{206}H_{176}Cr_7F_8NNiO_{40}$	$C_{251}H_{343.5}Cr_7F_8N_{19.5}NiO_{80}$	$C_{134}H_{128}Cr_7F_8NNiO_{32}$
Formula weight	3880.18	5488.64	2839.08
Temperature/K	150.00(10)	150.00(10)	100.00(10)
Crystal system	orthorhombic	tetragonal	monoclinic
Space group	$P2_12_12$	P4/ncc	C2/c
a/Å	31.9625(7)	26.7607(9)	24.0332(4)
b/Å	17.1553(5)	26.7607(9)	24.0911(5)
c/Å	16.9723(4)	40.561(3)	23.7630(6)
α/°	90	90	90
β/°	90	90	94.616(2)
γ/°	90	90	90
Volume/$Å^3$	9306.4(4)	29047(3)	13713.8(5)
Z	2	4	4
$\rho_{calc}g/cm^3$	1.385	1.255	1.375
μ/mm^{-1}	4.058	2.869	0.694
F(000)	4014.0	11576.0	5852.0
Crystal size/mm^3	0.278 × 0.254 × 0.129	0.325 × 0.278 × 0.215	0.19 × 0.187 × 0.054
Radiation	Cu Kα (λ = 1.54184)	Cu Kα (λ = 1.54184)	synchrotron (λ = 0.6889)
2Θ range for data collection/°	5.206 to 105.42	4.356 to 107.808	2.936 to 50.982
Index ranges	-32 ≤ h ≤ 31, -17 ≤ k ≤ 17, -16 ≤ l ≤ 16	-15 ≤ h ≤ 26, -25 ≤ k ≤ 24, -37 ≤ l ≤ 40	-30 ≤ h ≤ 30, -30 ≤ k ≤ 30, -29 ≤ l ≤ 29
Reflections collected	54143	75476	90883
Independent reflections	10232 [R_{int} = 0.0744, R_{sigma} = 0.0560]	8330 [R_{int} = 0.0647, R_{sigma} = 0.0400]	14008 [R_{int} = 0.0593, R_{sigma} = 0.0469]
Data/restraints/parameters	10232/4220/1272	8330/2210/897	14008/6300/1644
Goodness-of-fit on F^2	1.040	1.039	1.040
Final R indexes [I>=2σ (I)]	R_1 = 0.0679, wR_2 = 0.1793	R_1 = 0.0884, wR_2 = 0.2616	R_1 = 0.0694, wR_2 = 0.2195
Final R indexes [all data]	R_1 = 0.0924, wR_2 = 0.2000	R_1 = 0.1347, wR_2 = 0.2997	R_1 = 0.0844, wR_2 = 0.2340
Largest diff. peak/hole / e $Å^{-3}$	1.24/-0.35	0.53/-0.29	1.06/-0.41
Flack parameter	0.012(13)		

Code	$[NH_2{}^nPr_2]$[**13**]
Empirical formula	$C_{102}H_{88}Cr_7F_8N_9NiO_{48}$
Formula weight	2782.52
Temperature/K	99.95(10)
Crystal system	orthorhombic
Space group	Pnma
a/Å	26.8743(8)
b/Å	27.9440(6)
c/Å	16.9778(5)
α/°	90
β/°	90
γ/°	90
Volume/Å^3	12749.9(6)
Z	4
ρ_{calc}g/cm^3	1.450
μ/mm^{-1}	0.816
F(000)	5660.0
Crystal size/mm^3	0.55 × 0.289 × 0.17
Radiation	Mo Kα (λ = 0.71073)
2Θ range for data collection/°	5.676 to 57.022
Index ranges	-33 ≤ h ≤ 24, -37 ≤ k ≤ 31, -22 ≤ l ≤ 13
Reflections collected	38968
Independent reflections	14760 [R_{int} = 0.0502, R_{sigma} = 0.0594]
Data/restraints/parameters	14760/4228/1031
Goodness-of-fit on F^2	1.011
Final R indexes [I>=2σ (I)]	R_1 = 0.0529, wR_2 = 0.1430
Final R indexes [all data]	R_1 = 0.0781, wR_2 = 0.1595
Largest diff. peak/hole / e Å^{-3}	0.65/-0.60

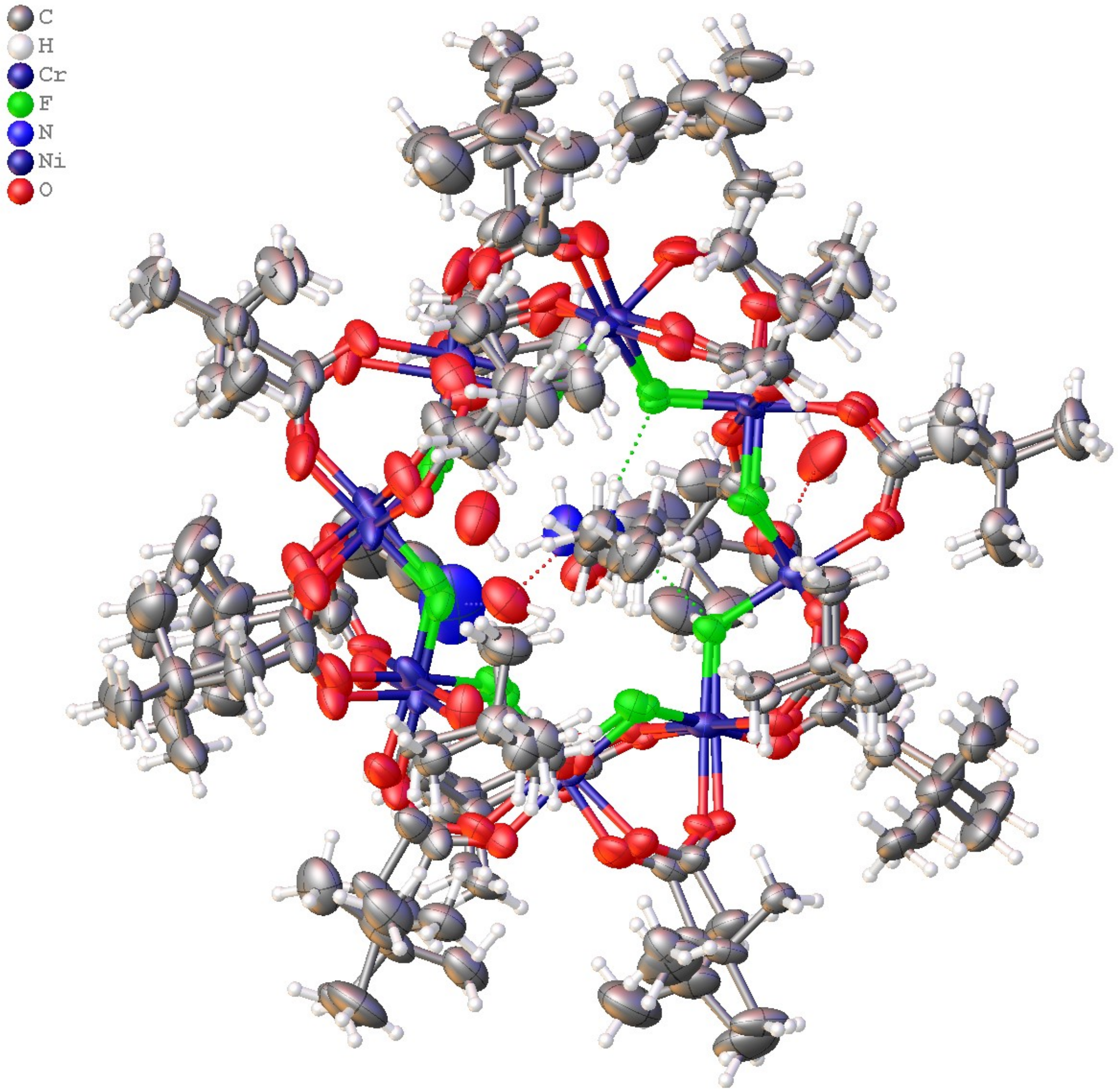

Figure S19: Crystal structure of [$NH_3{}^{n}Pr$][**3**]. Dark blue: chromium/nickel, green: fluorine, red: oxygen, blue: nitrogen, grey: carbon, white: hydrogen. Atomic displacement parameters are displayed with 50% probability.

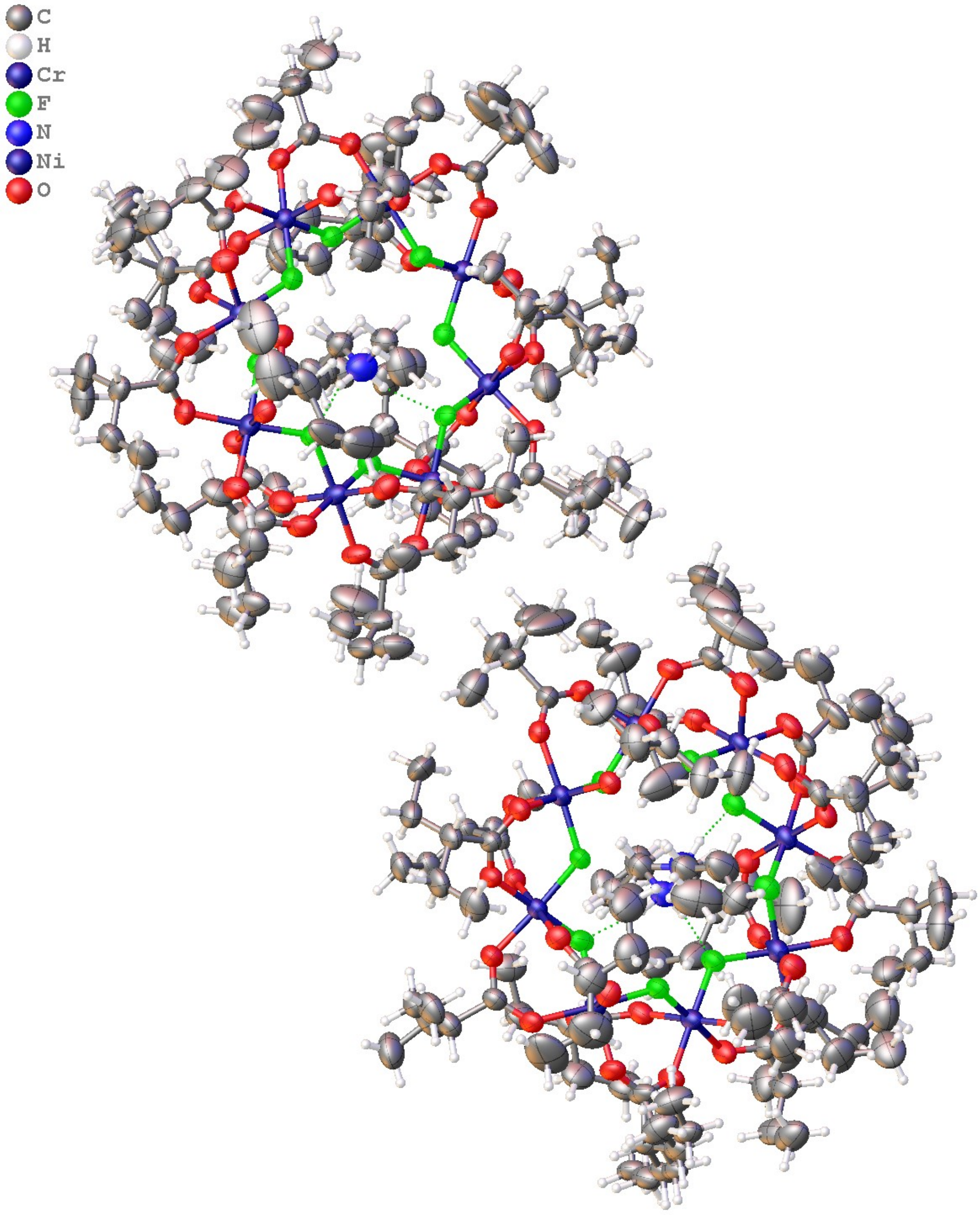

Figure S20: Crystal structure of $[NH_2(Allyl)_2]$[**5**]. Dark blue: chromium/nickel, green: fluorine, red: oxygen, blue: nitrogen, grey: carbon, white: hydrogen. Atomic displacement parameters are displayed with 50% probability.

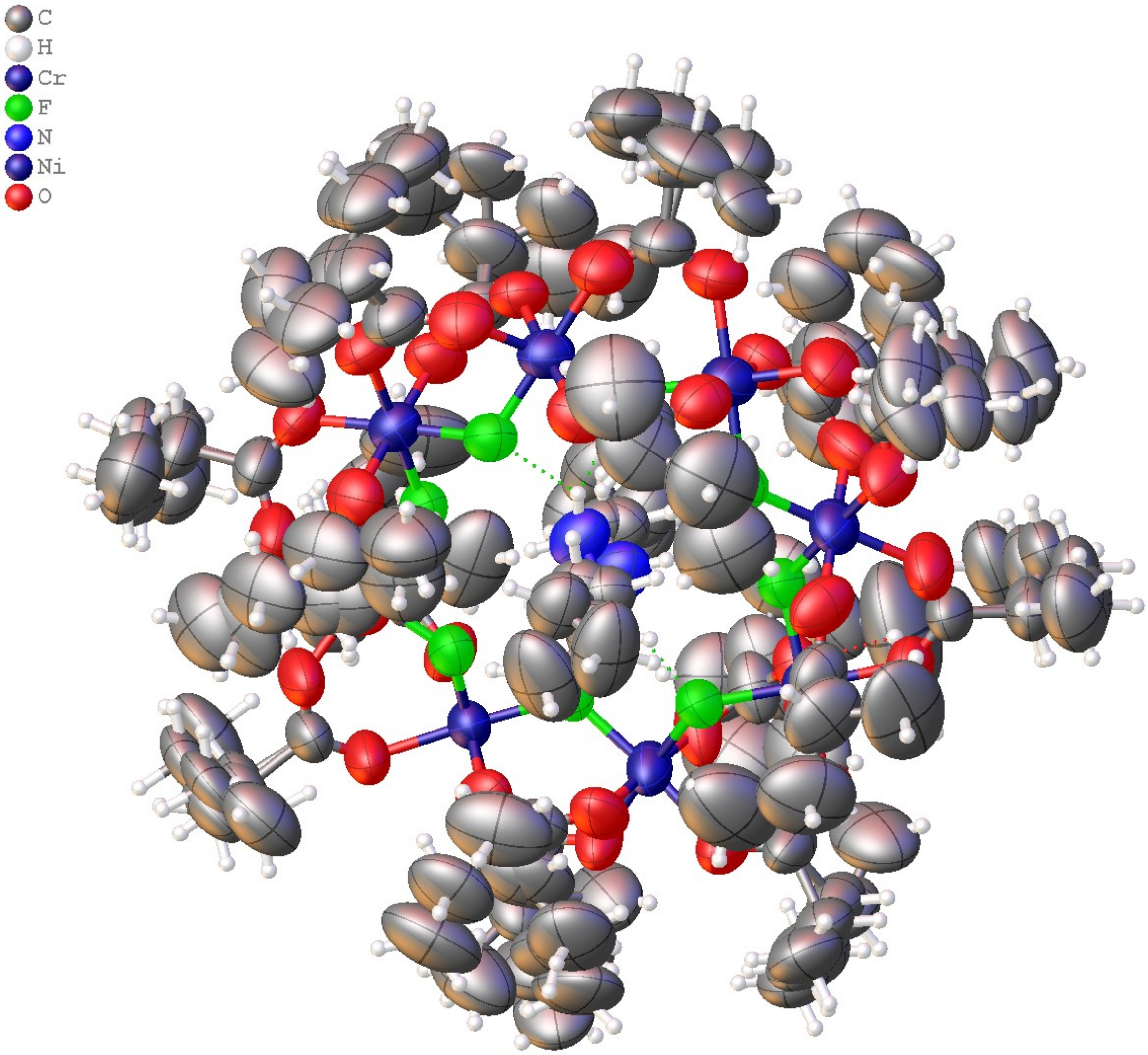

Figure S21: Crystal structure of [NH_2(Allyl)$_2$][**6**]. Dark blue: chromium/nickel, green: fluorine, red: oxygen, blue: nitrogen, grey: carbon, white: hydrogen. Atomic displacement parameters are displayed with 50% probability.

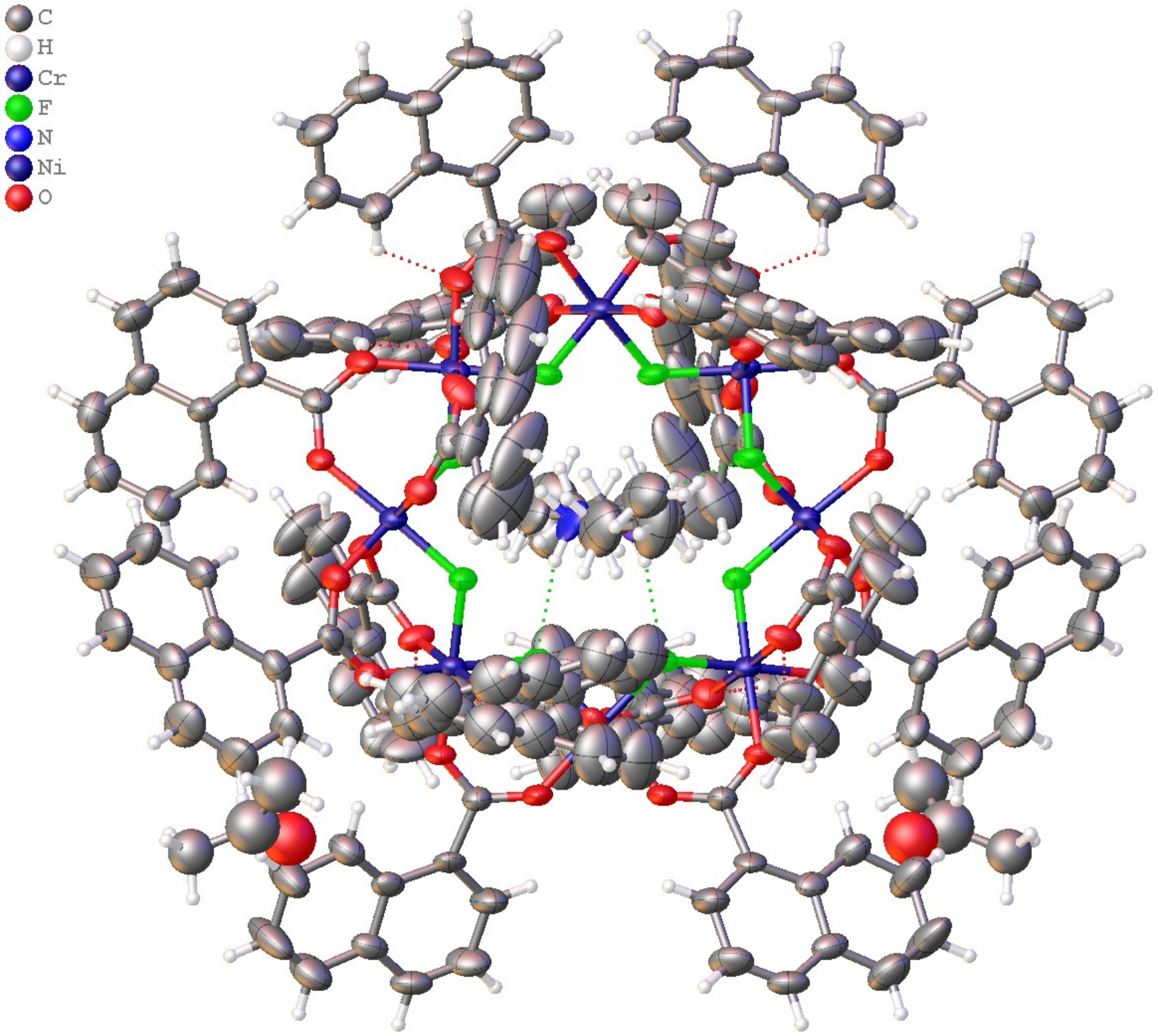

Figure S22: Crystal structure of [$NH_2{}^nPr_2$][**8**]. Dark blue: chromium/nickel, green: fluorine, red: oxygen, blue: nitrogen, grey: carbon, white: hydrogen. Atomic displacement parameters are displayed with 50% probability.

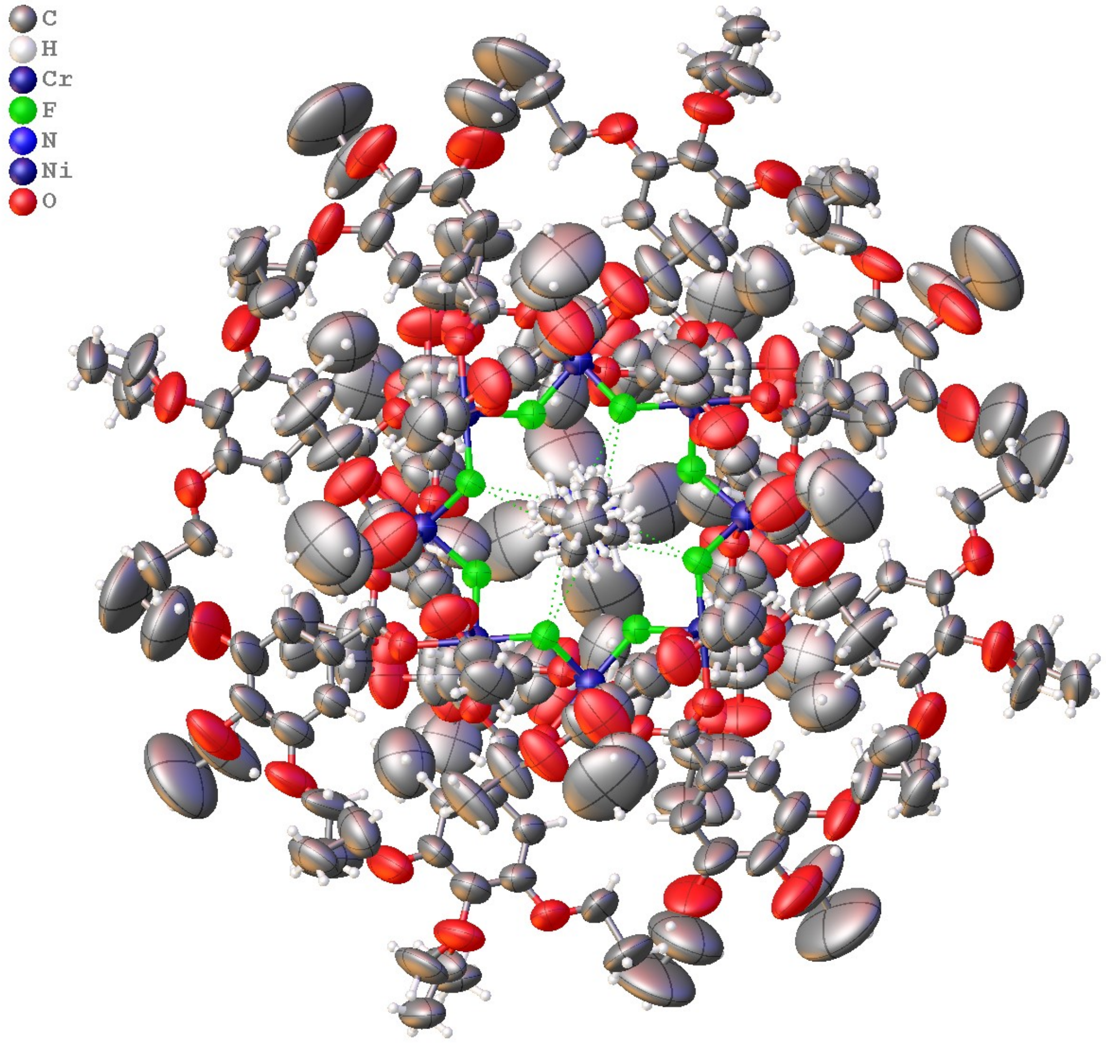

Figure S23: Crystal structure of $[NH_2{}^nPr_2]$[**10**]. Dark blue: chromium/nickel, green: fluorine, red: oxygen, blue: nitrogen, grey: carbon, white: hydrogen. Atomic displacement parameters are displayed with 50% probability.

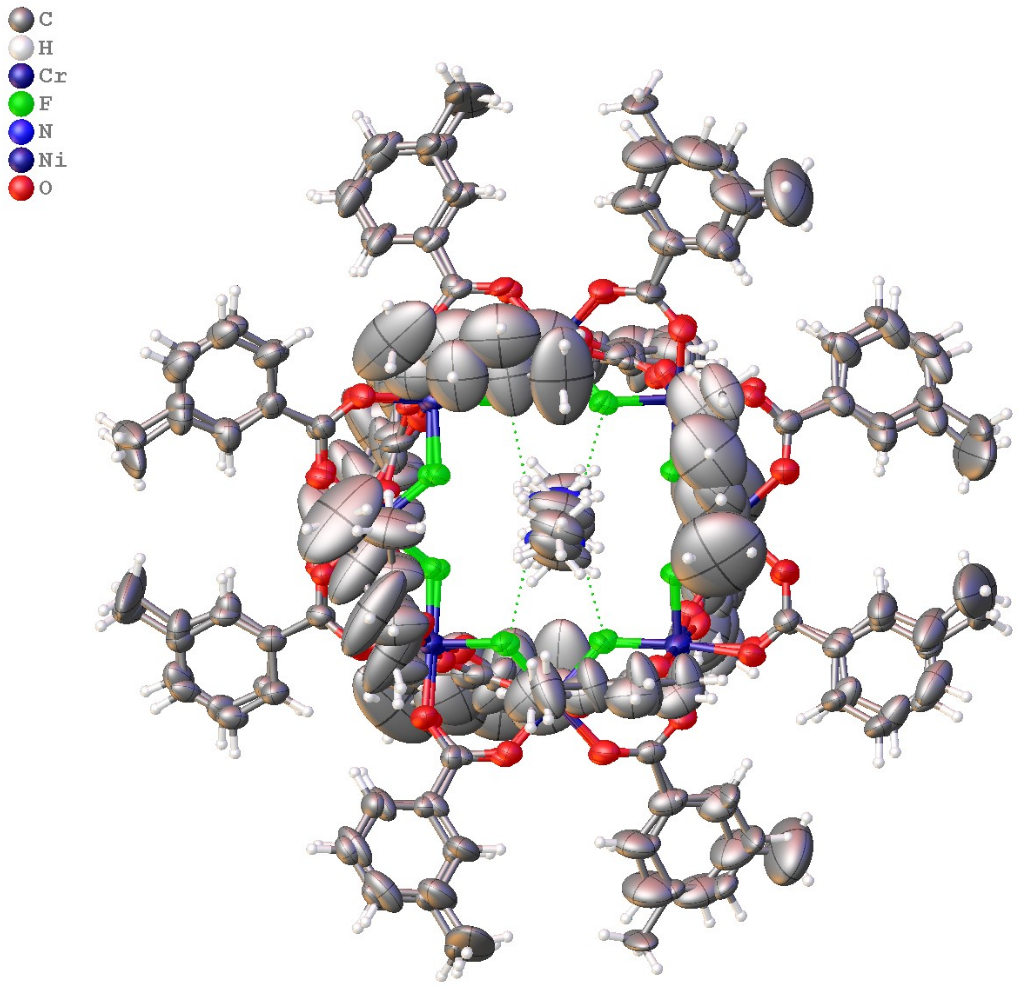

Figure S24: Crystal structure of $[NH_2{}^nPr_2]$[**11**]. Dark blue: chromium/nickel, green: fluorine, red: oxygen, blue: nitrogen, grey: carbon, white: hydrogen. Atomic displacement parameters are displayed with 50% probability.

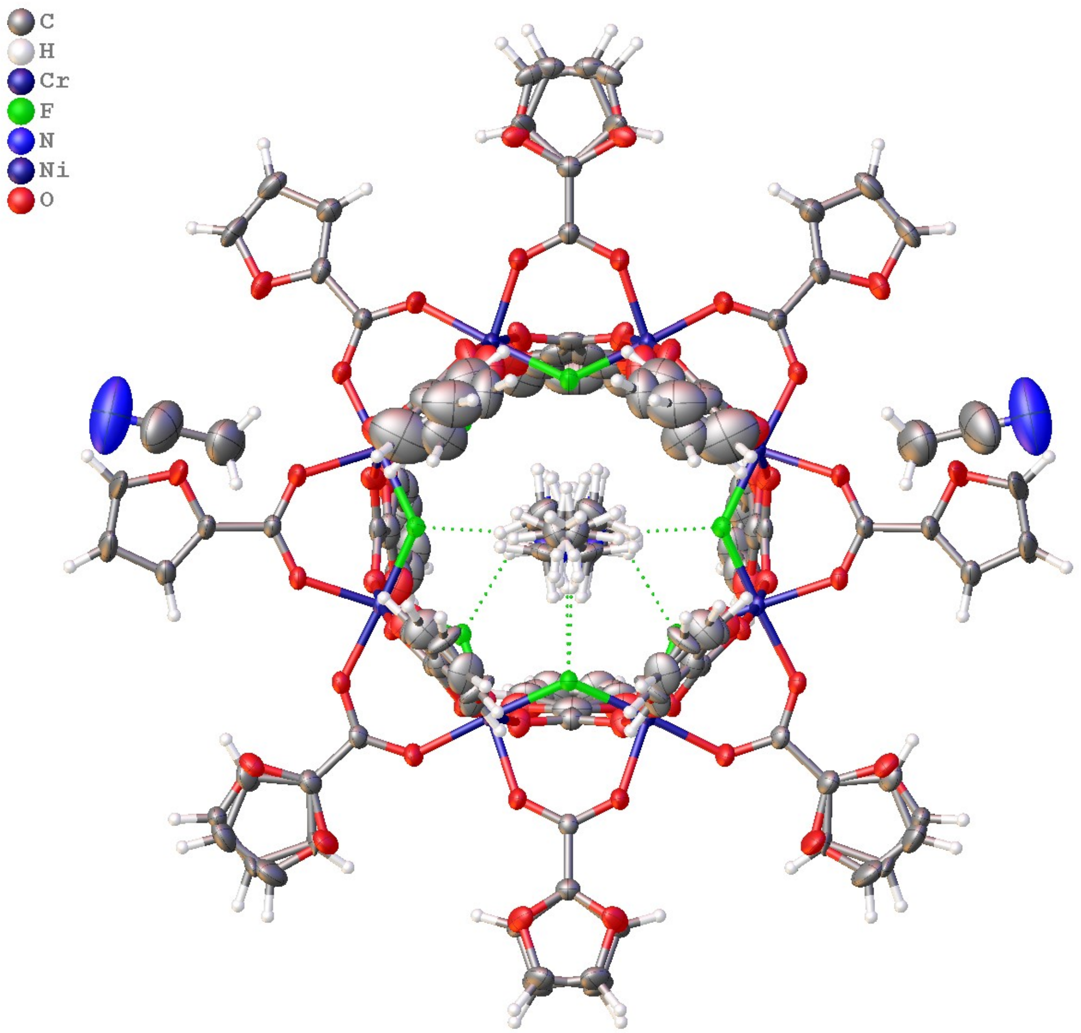

Figure S25: Crystal structure of [$NH_2{}^nPr_2$][**13**]. Dark blue: chromium/nickel, green: fluorine, red: oxygen, blue: nitrogen, grey: carbon, white: hydrogen. Atomic displacement parameters are displayed with 50% probability.

References

1 C. J. Wedge, G. A. Timco, E. T. Spielberg, R. E. George, F. Tuna, S. Rigby, E. J. L. McInnes, R. E. P. Winpenny, S. J. Blundell and A. Ardavan, *Phys. Rev. Lett.*, 2012, **108**, 107204.

2 F. K. Larsen, E. J. L. McInnes, H. E. Mkami, J. Overgaard, S. Piligkos, G. Rajaraman, E. Rentschler, A. A. Smith, G. M. Smith, V. Boote, M. Jennings, G. A. Timco and R. E. P. Winpenny, *Angew. Chem. Int. Ed.*, 2003, **42**, 101–105.

3 A. Ghirri, V. Corradini, C. Cervetti, A. Candini, U. del Pennino, G. Timco, R. J. Pritchard, C. A. Muryn, R. E. P. Winpenny and M. Affronte, *Adv. Funct. Mater.*, 2010, **20**, 1552–1560.

4 R. H. Laye, F. K. Larsen, J. Overgaard, C. A. Muryn, E. J. L. McInnes, E. Rentschler, V. Sanchez, S. J. Teat, H. U. Güdel, O. Waldmann, G. A. Timco and R. E. P. Winpenny, *Chem. Commun.*, 2005, 1125–1127.

5 D. Kaminski, A. L. Webber, C. J. Wedge, J. Liu, G. A. Timco, I. J. Vitorica-Yrezabal, E. J. L. McInnes, R. E. P. Winpenny and A. Ardavan, *Phys. Rev. B*, 2014, **90**, 184419.

6 H. Nowell, S. A. Barnett, K. E. Christensen, S. J. Teat and D. R. Allan, *J. Synchrotron Radiat.*, 2012, **19**, 435–441.

7 D. R. Allan, H. Nowell, S. A. Barnett, M. R. Warren, A. Wilcox, J. Christensen, L. K. Saunders, A. Peach, M. T. Hooper, L. Zaja, S. Patel, L. Cahill, R. Marshall, S. Trimnell, A. J. Foster, T. Bates, S. Lay, M. A. Williams, P. V. Hathaway, G. Winter, M. Gerstel and R. W. Wooley, *Crystals*, 2017, **7**, 336.

8 Rigaku Oxford Diffraction (version 1.171.43.XX) Rigaku Corporation, Wroclaw, Poland 2025.

9 G. M. Sheldrick, *Acta Crystallogr. A*, 2015, **71**, 3–8.

10 G. M. Sheldrick, *Acta Crystallogr. Sect. C Struct. Chem.*, 2015, **71**, 3–8.

11 K. L. Fort, M. Van De Waterbeemd, D. Boll, M. Reinhardt-Szyba, M. E. Belov, E. Sasaki, R. Zschoche, D. Hilvert, A. A. Makarov and A. J. R. Heck, *Analyst*, 2018, **143**, 100–105.

12 N. Geue, T. S. Bennett, A.-A.-M. Arama, L. A. I. Ramakers, G. F. S. Whitehead, G. A. Timco, P. B. Armentrout, E. J. L. McInnes, N. A. Burton, R. E. P. Winpenny and P. E. Barran, *J. Am. Chem. Soc.*, 2022, **144**, 22528–22539.

13 N. Geue, T. S. Bennett, L. A. I. Ramakers, G. A. Timco, E. J. L. McInnes, N. A. Burton, P. B. Armentrout, R. E. P. Winpenny and P. E. Barran, *Inorg. Chem.*, 2023, **62**, 2672–2679.

14 N. Geue, G. A. Timco, G. F. S. Whitehead, E. J. L. McInnes, N. A. Burton, R. E. P. Winpenny and P. E. Barran, *Nat. Synth.*, 2023, **2**, 926–936.

15 T. S. Bennett, N. Geue, G. A. Timco, G. F. S. Whitehead, I. J. Vitorica-Yrezabal, P. E. Barran, E. J. L. McInnes and R. E. P. Winpenny, *Chem. – Eur. J.*, 2024, **30**, e202400432.

16 F. Neese, *WIREs Comput. Mol. Sci.*, 2012, **2**, 73–78.

17 F. Neese, F. Wennmohs, U. Becker and C. Riplinger, *J. Chem. Phys.*, 2020, **152**, 224108.

18 T. Lu, *J. Chem. Phys.*, 2024, **161**, 082503.

19 E. R. Johnson, S. Keinan, P. Mori-Sánchez, J. Contreras-García, A. J. Cohen and W. Yang, *J. Am. Chem. Soc.*, 2010, **132**, 6498–6506.

20 M. Friede, S. Ehlert, S. Grimme and J.-M. Mewes, *J. Chem. Theory Comput.*, 2023, **19**, 8097–8107.

21 N. Mardirossian and M. Head-Gordon, *J. Chem. Phys.*, 2016, **144**, 214110.

22 E. Caldeweyher, S. Ehlert, A. Hansen, H. Neugebauer, S. Spicher, C. Bannwarth and S. Grimme, *J. Chem. Phys.*, 2019, **150**, 154122.

23 F. Weigend, *Phys. Chem. Chem. Phys.*, 2006, **8**, 1057–1065.

24 F. Weigend and R. Ahlrichs, *Phys. Chem. Chem. Phys.*, 2005, **7**, 3297–3305.

25 S. Grimme, *Chem. – Eur. J.*, 2012, **18**, 9955–9964.

26 A. V. Marenich, C. J. Cramer and D. G. Truhlar, *J. Phys. Chem. B*, 2009, **113**, 6378–6396.
27 D. G. Liakos, M. Sparta, M. K. Kesharwani, J. M. L. Martin and F. Neese, *J. Chem. Theory Comput.*, 2015, **11**, 1525–1539.
28 F. Neese and E. F. Valeev, *J. Chem. Theory Comput.*, 2011, **7**, 33–43.
29 F. Neese, F. Wennmohs, A. Hansen and U. Becker, *Chem. Phys.*, 2009, **356**, 98–109.
30 G. L. Stoychev, A. A. Auer and F. Neese, *J. Chem. Theory Comput.*, 2017, **13**, 554–562.
31 S. Grimme, A. Hansen, S. Ehlert and J.-M. Mewes, *J. Chem. Phys.*, 2021, **154**, 064103.
32 O. A. Vydrov and T. Van Voorhis, *J. Chem. Phys.*, 2010, **133**, 244103.
33 P. Muller, *Pure Appl. Chem.*, 1994, **66**, 1077–1184.
34 S. Sastre, R. Casasnovas, F. Muñoz and J. Frau, in *8th Congress on Electronic Structure: Principles and Applications (ESPA 2012): A Conference Selection from Theoretical Chemistry Accounts*, eds. J. J. Novoa and M. F. Ruiz López, Springer, Berlin, Heidelberg, 2014, pp. 51–58.
35 W. M. Haynes, Ed., *CRC Handbook of Chemistry and Physics*, CRC Press, Boca Raton, 97th edn., 2016.
